# Catalytic Behavior of Cobalt Complexes Bearing Pyridine–Oxime Ligands in Isoprene Polymerization

**DOI:** 10.3390/polym15244660

**Published:** 2023-12-10

**Authors:** Yuanxu Du, Shuo Gao, Hui Ma, Siqi Lu, Zhenhua Zhang, Mengmeng Zhao

**Affiliations:** 1 School of Chemistry and Chemical Engineering, Linyi University, Linyi 276000, China; 202226100109@lyu.edu.cn (Y.D.); gaoshuo9802@163.com (S.G.); maroumodisi@163.com (H.M.); 17866937745@163.com (S.L.); 2 College of Agriculture and Forestry, Linyi University, Linyi 276000, China

**Keywords:** cobalt(II) catalyst, pyridine-2-aldoxime ligand, isoprene polymerization, low cocatalyst consumption, high catalytic activity

## Abstract

Several cobalt(II) complexes **Co1**–**Co3** bearing pyridine–oxime ligands (**L1** = pyridine-2-aldoxime for **Co1**; **L2** = 6-methylpyridine-2-aldoxime for **Co2**; **L3** = phenyl-2-pyridylketoxime for **Co3**) and picolinaldehyde O-methyl oxime (**L4**)-supported **Co4** were synthesized and well characterized by FT-IR, mass spectrum and elemental analysis. The single-crystal X-ray diffraction of complex **Co2** reveals that the cobalt center of CoCl_2_ is coordinated with two 6-methylpyridine-2-aldoxime ligands binding with *N*_pyridine_ and *N*_oxime_ atoms, which feature a distorted octahedral structure. These Co complexes **Co1**–**Co4** displayed extremely high activity toward isoprene polymerization upon activation with small amount of AlClEt_2_ in toluene, giving polyisoprene with high activity up to 16.3 × 10^5^ (mol of Co)^−1^(h)^−1^. And, the generated polyisoprene displayed high molecular weights and narrow molecular distribution with a *cis*-1,4-enriched selectivity. The type of cobalt complexes, cocatalyst and reaction temperature all have effects on the polymerization activity but not on the microstructure of polymer.

## 1. Introduction

The development of novel catalysts for diene polymerization with high activity and selectivity has attracted considerable attention in the past decades for improved synthetic rubber [1,2]. Isoprene is one of the most popular monomers, playing an increasingly important role in the synthetic rubber industry ever since being isolated from natural rubber by Williams in 1860. The stereospecific polymerization of isoprene allows the following stereoregular polymers to be given, including *cis*-1,4-, *trans*-1,4-, 3,4-, 1,2-unit, which exhibit different mechanical properties and application direction due to the otherness of chain parameters. For example, *cis*-1,4- polyisoprene has a microstructure similar to natural rubber and can be used as an alternative to natural rubber. The 3,4- polyisoprene is generally applied in the tire industry to improve the wet skid resistance of tread stock. Hence, developing well-performed *cis*-1,4-*alt*-3,4- polyisoprene materials with various component contents and sequence structure is one of the main goals of rubber industry [3,4].

Transition metal (Sc, Nd, Ti, V, Cr, Fe, Co, Ni, Pd, etc.)-catalyzed diene polymerization is a successful technique invention, and has achieved a significant breakthrough for the field of synthetic rubber with good performances [5,6,7,8]. Ever since the Ziegler–Natta catalysts were invented, the research has mainly focused on simple metal salts (Ti [9], Nd [10], and Co [11] with halide, acetonate, etc.) in combination with alkyl aluminum. To improve the activity of the transition metal catalyst, a variety of ligands containing pyridine, imine, phosphine and phenolate moieties have been applied to modify the electronic and steric environment at the metal’s center. And, it has been proven that the ancillary ligand plays an important role in the catalytic process and polymer properties [12,13,14,15,16,17,18]. Therefore, much research has been focused on cobalt catalyst, which is known as the most versatile catalyst among the various transition metals, and has characteristics of low cost, high stability and easy preparation. And, these cobalt catalysts show good catalytic effects in regulating polyolefin microstructure, molecular weight and molecular weight distribution. At first, several cobalt salts (e.g., CoCl_2_ and Co(acac)_2_) combined with suitable alkylating agents were used to catalyze diene polymerization with disappointing catalytic effects [6,19,20,21]. Subsequently, efforts to develop well-defined cobalt complexes to catalyze the polymerization of conjugated dienes were made. Many kinds of monodentate, bidentate, tridentate and tetradentate ligands with phosphine, nitrogen, oxygen, and sulfur atoms were applied for the synthesis of cobalt catalysts [8,22]. For example, researchers started using phosphorus ligands to synthesize cobalt complexes, which demonstrated the desired management of microstructure and molecular weight in the polymerization of butadiene, isoprene and 1,3-hexadienes in combination with an Al cocatalyst [3,23,24,25,26,27,28]. By skillfully altering the ligand structure and ligating atom, the cobalt catalysts were able to exhibit high activity, and a wide variety of polymers with different properties and structures were available. Further work on polymerization of diene catalyzed by cobalt complexes with tridentate ligands or tetradentate ligand containing a *N* atom and other *O*, *P* or *S* heteroatoms (*N-N-N* [29,30,31,32,33,34,35,36,37,38,39], *N-N-O* [40,41,42,43], *N-N-S* [44], *N-N-P* [45,46,47] and *N-N-O-O* [48], etc.) have been reported. For instance, Gong and his coworkers synthesized a novel type of hemilabile donor ligated cobalt dichloride complexes (*N*,*N*,*P*/*O*/*S*), and the nature of the hemilabile donor (*P*, *O* and *S*) determines the coordination chemistry of the complexes, which demonstrated the high activity and enriched cis-1,4- selectivity of polyisoprene. The kinetic studies reveal that the catalyst systems follow a typical living mechanism [46]. Meanwhile, *N*,*N*-containing bidentate ligands, such as iminopyridine and α-diimine, have also been tested for cobalt-catalyzed polymerization, and attracted extensive investigations in the past few years [49,50,51,52,53,54,55,56,57,58]. For example, Sun and coworkers reported a series of (8-(arylimino)-5,6,7-trihydroquinolin-2-yl)methyl acetate (*N*,*N*) ligated cobalt complexes and applied them in isoprene polymerization. This catalyst system demonstrated a high activity of 1.37 × 10^5^ (mol of Co)^−1^(h)^−1^ to give polyisoprene with high molecular weight and the *cis*-1,4 configuration nearly to 70%.

Due to our ongoing burning desire to develop polymerization catalysts with high performance, we are interested in transition metal catalysts coordinating with N,N-bidentate ligands for diene polymerization [58,59,60,61,62,63,64]. In a recent piece of work, we reported that the pyridine–oxime ligand exhibited high performance in iron catalyzed isoprene polymerization with very low amount of methylaluminoxane [65]. Confirming the fundamental role played by the nature of the pyridine–oxime ligand and the type of cobalt catalyst in determining the catalytic activity and selectivity [66,67], herein we describe a series of pyridine–oxime ligated cobalt catalysts and study their catalytic properties for isoprene polymerization.

## 2. Materials and Methods

### 2.1. General Considerations

All experiments were carried out under argon atmosphere by using standard Schlenk techniques or in glovebox. CoCl_2_ (Macklin Biochemical Co., Ltd., Shanghai, China) and diethylaluminum chloride (AlEt_2_Cl, 2.0 M solution in hexane, Macklin Biochemical Co., Ltd., Shanghai, China) were purchased and used without further purification. Toluene (Sinopharm Chemical Reagent, Shanghai, China) was refluxed over sodium and distilled and stored over molecular sieves under nitrogen. Hexane and dichloromethane (Sinopharm Chemical Reagent, Shanghai, China) were refluxed over calcium hydride and distilled and stored over molecular sieves in glove box. Isoprene, picolinaldehyde, MeOH, Na_2_CO_3_ and Na_2_SO_4_ (Sinopharm Chemical Reagent, Shanghai, China) was purchased and freshly distilled over calcium hydride under nitrogen atmosphere.

### 2.2. Co(II) Complexes Preparation

Ligands **L1** and **L3** were purchased and used directly without treatment. The ligands **L2** and **L4** were synthesized and characterized according to the previous report [65]. A typical procedure for the synthesis of ligand was carried out as follows. To a suspension of picolinaldehyde (1.0 equiv.) in MeOH, the corresponding amine (1.0 equiv.) was added, and followed by Na_2_CO_3_ (0.5 equiv.). The mixture was stirred at room temperature for 4 h. Then, the suspension was filtered and the solution was transferred to a separatory funnel, washed with water (3 × 20 mL) and dried over anhydrous Na_2_SO_4_. The solvent was removed in the rotary evaporator and the product was dried under vacuum to give ligand. All the ^1^H NMR and ^13^C NMR of ligands were collected on a Bruker Avance III 400 MHz instrument (Bruker, Karlsruhe, Germany) at 298K, using tetramethyl silane (TMS; CIL, Andover, MA, USA) as internal standard.

**L2**: yellow solid, 2.1 g, 90% yield; ^1^H NMR (400 MHz, CDCl_3_, 298 K) δ 8.27–8.24 (m, 2H), 7.63–7.54 (m, 2H), 7.14 (m, 1H), 2.58 (s, 3H); ^13^C NMR (100 MHz, CDCl_3_, 298 K) δ 158.5, 151.0, 150.9, 136.7, 123.8, 118.2, 24.3.

**L4**: light yellow oil, 3.1 g, 89% yield; ^1^H NMR (400 MHz, CDCl_3_, 298 K) δ 8.61–8.59 (m, 1H), 8.15 (s, 1H), 7.77 (dt, *J* = 8.0, 1.2 Hz, 1H), 7.68 (dt, *J* = 7.7, 1.8 Hz, 1H), 7.24 (m, 1H), 4.02 (s, 3H). ^13^C NMR (100 MHz, CDCl_3_, 298 K) δ 151.5, 149.5, 149.0, 136.2, 123.9, 121.0, 62.4.

All the cobalt complexes **Co1**–**Co4** were synthesized using the following methods. Into a 25 mL flask, CoCl_2_ (1 equiv.) and 8 mL CH_2_Cl_2_ in glove box were added, and the ligand in 2 mL CH_2_Cl_2_ was added dropwise. The resultant mixture was stirred at room temperature overnight. The solvent was removed under vacuum to 5 mL and the complex was precipitated by addition of hexane (3 mL). The precipitation was collected by filtration and washed three times with hexane (3 × 5 mL) and dried under vacuum to produce Co(II) complex. Mass spectra for cobalt complexes were detected using ACQUITYTM UPLC & Q-TOF MS Premier (Waters, Milford, MA, USA) at Shanghai Jiao Tong University (Shanghai, China). Elemental analysis was recorded using Vario EL III elemental analyzer (Elementar Corporation, Hanau, Germany) at Shanghai Institute of Organic Chemistry (Shanghai, China). X-ray diffraction data was obtained on Smart 1000 diffractometer with Mo K-alpha X-ray source (λ = 0.71073 Å) at 298 K (Bruker, Karlsruhe, Germany) at Liaocheng university (Shandong, China). Attenuated total reflection–infrared (ATR-IR) spectroscopy was conducted using Thermo Scientific Nicolet iN10 (Thermo Fisher Scientific, Waltham Mass, America) at Shanghai Jiao Tong University (Shanghai, China).

**Co1**: CoCl_2_ (1 equiv.) and ligand **L1** (2 equiv.) were conducted to give jade-green solid. A total of 359 mg, 83% yield. ATR-IR (cm^−1^): 3064, 1640, 1602, 1478, 1450, 1303, 1254, 1037, 1014, 949, 886, 774. TOF-MS-ES+ (*m*/*z*): calcd. For [C_12_H_12_ClCoN_4_O_2_**·**H_2_O**·**3CH_3_CN]^+^: 481.0853, found: 480.9859. Anal.: calcd. For C_12_H_12_Cl_2_CoN_4_O_2_: C, 38.53; H, 3.23; N, 14.98; found: C, 38.34; H, 3.23; N, 14.81.

**Co2**: CoCl_2_ (1 equiv.) and ligand **L2** (2 equiv.) were conducted to give orange solid. A total of 421 mg, 87% yield. ATR-IR (cm^−1^): 3055, 1649, 1601, 1493, 1458, 1317, 1255, 1046, 1005, 953, 802, 695. TOF-MS-ES+ (*m*/*z*): calcd. For [C_14_H_16_ClCoN_4_O_2_**·**3MeOH**·**3CH_3_CN]^+^: 555.1585, found: 555.0580. Anal.: calcd. For C_14_H_16_Cl_2_CoN_4_O_2_: C, 41.81; H, 4.01; N, 13.93; found: C, 41.96; H, 4.02; N, 13.92.

**Co3**: CoCl_2_ (1 equiv.) and ligand **L3** (2 equiv.) were conducted to give green solid. A total of 272 mg, 86% yield. ATR-IR (cm^−1^): 3064, 1640, 1602, 1478, 1450, 1303, 1254, 1037, 1014, 949, 886, 774, 717. TOF-MS-ES+ (*m*/*z*): calcd. for [C_12_H_10_ClCoN_2_O**·**2MeOH**·**2CH_3_CN]^+^: 438.0863, found: 438.0762. Anal.: calcd. For C_24_H_20_Cl_2_CoN_4_O_2_: C, 54.77; H, 3.83; N, 10.65; found: C, 54.21; H, 4.02; N, 10.39.

**Co4**: CoCl_2_ (1 equiv.) and ligand **L4** (2 equiv.) were conducted to give purple solid. A total of 257 mg, 83% yield. ATR-IR (cm^−1^): 1655, 1569, 1522, 1356, 1274, 1023, 930, 771. TOF-MS-ES+ (*m*/*z*): calcd. for [C_7_H_8_ClCoN_2_O**·**2MeOH**·**CH_3_CN]^+^: 335.0441, found: 335.0557. Anal.: calcd. For C_14_H_16_Cl_2_CoN_4_O_2_: C, 41.81; H, 4.01; N, 13.93; found: C, 41.75; H, 4.00; N, 13.79.

### 2.3. Procedure for Isoprene Polymerization

The isoprene polymerization was carried out in a 25 mL Schlenk reactor by using toluene as solvent. In a universal method, the reactor was pumped and inflated three times. The cobalt complex was weighed in glove box and then added into a Schlenk reactor. The required amount of solvent, isoprene and cocatalyst was sequentially added into the Schlenk reactor under argon atmosphere outside of the glove box. At the specified time, the polymerization was quenched with HCl solution in methanol (methanol/HCl = 50/1). Polymer was recovered in methanol with an antioxidant 2,6-di–tert–butyl–4-methylphenol (BHT, 0.5 wt.%). The polymer was collected by filtration and washed with ethanol for several times and dried under vacuum at 50 °C until no change was observed in weight. The polymer yields were determined by gravimetry.

### 2.4. Polymer Characterizations

The polymer structure was analyzed by NMR spectra conducting on a Bruker Advance 400 spectrometer at 298 K. ^1^H NMR (400 Hz, CDCl_3_) and ^13^C NMR spectra (100 Hz, CDCl_3_) of polyisoprene were recorded by using trimethylsilane as internal reference. The polyisoprene microstructure of the 1,4 and 3,4 ratio was determined from ^1^H NMR of the 1,4 =CH signals at 5.15 ppm and the 3,4 =CH_2_ signal at 4.7 ppm. The *trans*/*cis*-1,4 stereoisomer ratio was distinguished from ^13^C NMR of the –CH_3_ signals at 23.8 ppm (for *cis*-1,4) and at 16.3 ppm (for *trans*-1,4). The molecular weights (*M_n_*) and molecular weight distributions (*M_w_*/*M_n_*) of polymers were measured using gel permeation chromatography (GPC) using a PL-GPC 50 chromatography and maintained at 25 °C by using THF as eluent and polystyrene as standard.

## 3. Results and Discussions

### 3.1. Synthesis and Characterization of Co(II) Complexes

**Scheme 1:**
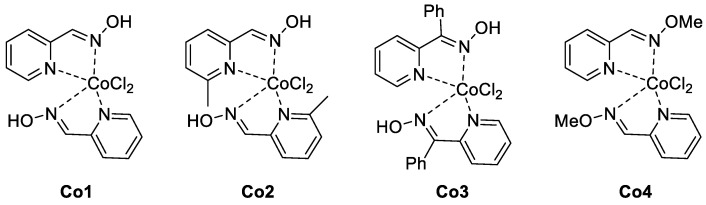
The structures of the precursors employed.

**Figure 1:**
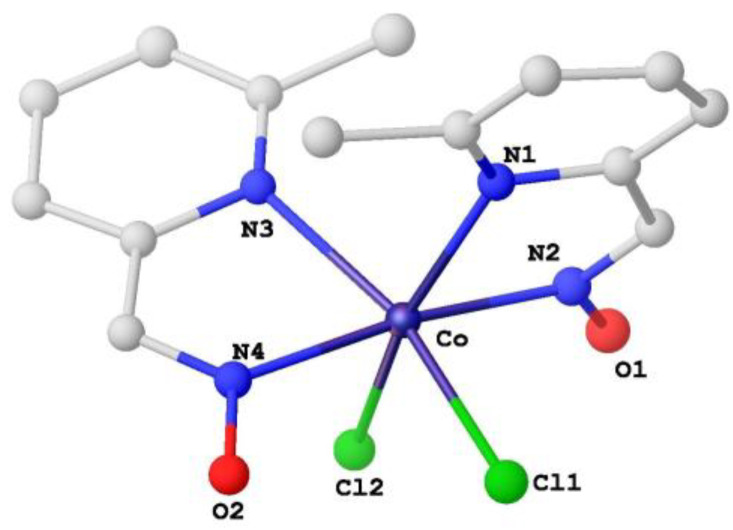
Molecular structure of **Co2** (hydrogen atoms were omitted for clarity). Selected bond distances (Å) and angles (deg): Co-N(1) 2.188(17), Co-N(2) 2.098(16), Co-N(3) 2.219(16), Co-N(4) 2.132 (16), Co-Cl(1) 2.470(7), Co-Cl(2) 2.455(6), O(1)-H(1) 0.8200, O(2)-H(2) 0.8200, N(2)-Co-N(4) 172.1(5), N(2)-Co-N(1) 77.2(6), N(4)-Co-N(1) 109.6(6), N(2)-Co-N(3) 109.5(6), N(4)-Co-N(3) 74.7(6), N(1)-Co-N(3) 94.0(4), Cl(2)-Co-Cl(1) 98.68(15). The gray sphere represents the carbon atom, and the hydrogen atom is omitted.

The Co(II) complexes were synthesized by the prepared ligands coordinating with cobalt chloride in anhydrous CH_2_Cl_2_ under argon atmosphere to provide the targeted complexes **Co1**–**Co4** with high yields. All the cobalt complexes performed well in characterization analysis, including ATR-IR, high resolution mass spectroscopies and elemental analysis (Scheme 1). Single crystals of complex **Co2** were obtained by diffusing hexane into its saturated dichloromethane solution at −30 °C in glovebox, and have been deposited in CCDC with codes number of 2160527. As seen in the structure shown in Figure 1 and Tables S1–S3, the **Co2** complex adopted a distorted octahedron coordination geometry surrounded by two **L2** ligands and one CoCl_2_ molecule, wherein two *N*_pyridine_ atoms (N1 and N3) and two chloride atoms formed the basal equatorial plane and the angle of the axis N2-Co-N4 is 172.1°. Moreover, the *N*_oxime_ atoms of each ligand have a stronger coordination than *N*_pyridine_ at the cobalt center according to the bond lengths [Co–N1 = 2.188(17), Co–N2 = 2.098(16), Co–N3 = 2.219(16), Co–N4 = 2.132(16)]. Consistent with the previous work of pyridine–oxime iron complex [65], the oxime proton is unionized.

### 3.2. Polymerization of Isoprene Catalyzed by Co(II) Complexes

Initial screening for isoprene polymerization was concentrated on the type of catalyst to identify whether there were some differences between N-OH group and N-OMe group. Complexes **Co1** and **Co4** were used as precatalysts activated by AlEt_2_Cl in toluene solution (5 mL) under various temperatures, as summarized in Table 1. The results revealed that the complex **Co1** converted the monomer to polyisoprene with high activity under the condition of [Co]/[isoprene]/[AlEt_2_Cl] = 1/2000/500, giving a polymerization yield of 95% at 25 °C within 10 min. The produced polyisoprene has a structure with 69% of *cis*-1,4 and 31% of 3,4 units and a molecular weight of 1.2 × 10^5^ g/mol with low molecular weight distribution of 1.6 (entry 1, Table 1). In addition, complex **Co4** showed a similar manner of polymerization with a lower activity, with 6.8 × 10^5^ g (mol of Co)^−1^(h)^−1^, than **Co1,** with 7.7 × 10^5^ g (mol of Co)^−1^(h)^−1^ (entry 2, Table 1). Changing the reaction temperature did have effects on reactivity and selectivity. At 70 °C, the complex **Co1** could lead to a polyisoprene yield higher than 83%, with high thermostability when the reaction was stopped at 10 min (entry 3, Table 1). But for complex **Co4**, the reaction activity decreased from 6.8 × 10^5^ g (mol of Co)^−1^(h)^−1^ at 25 °C to 4.6 × 10^5^ g (mol of Co)^−1^(h)^−1^ at 70 °C (entry 4, Table 1). And, the above results may be connected to the fact that the N-OH moiety displayed a higher catalytic activity and higher heat stability than the N-OMe group under the same current conditions. However, when the polymerization reactions were conducted at −30 °C, no polyisoprene could be produced for both complexes **Co1** and **Co4** (entries 5 and 6, Table 1). In addition, significant composition drift is not observed for the resultant isoprene for complexes Co1 and Co4 with enriched *cis*-1,4 motif. And, the *cis*-1,4 units content slightly decrease at high temperature.

**Figure 2:**
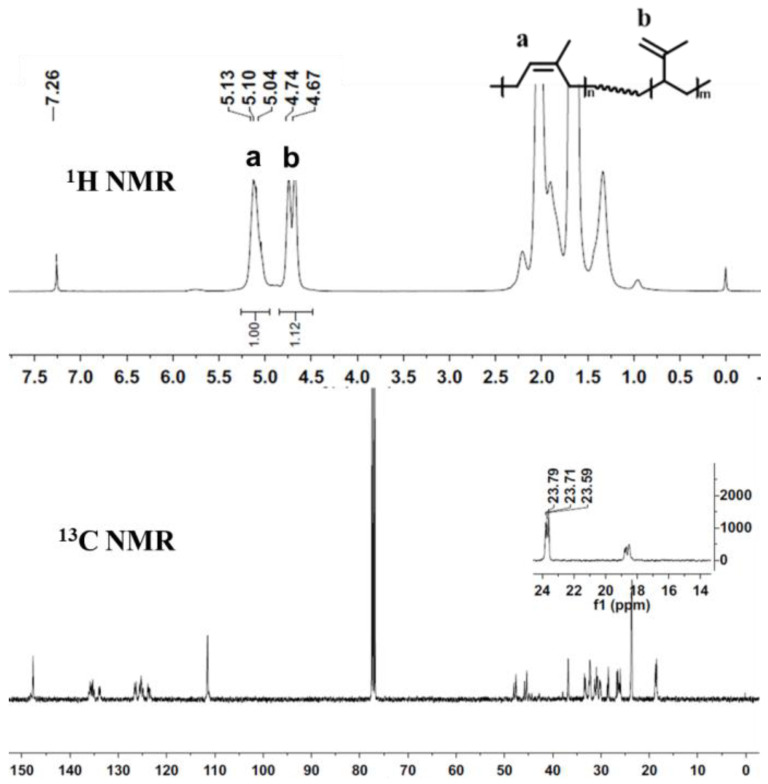
^1^H and ^13^C NMR spectra of the representative sample of polyisoprene obtained at [Co]/[AlEt_2_Cl] = 1/10 (Table 2, entry 2). ^1^H NMR of the 1,4 =CH signals at **a** and the 3,4 =CH_2_ signal at **b**.

Subsequently, complexes **Co1** catalyzed isoprene polymerization with various cocatalyst feeding amounts and reaction time were summarized, as seen in Table 2. For complex **Co1**, changing the amount of [Co]/[AlEt_2_Cl] from 1/500 to 1/50 (entry 1, Table 2) did not vary reactivity significantly when the reaction went on for 10 min, and also gave >99% yield of polyisoprene. And, complete conversion of isoprene could be achieved even though the [Co]/[AlEt_2_Cl] is changed to 1/10 (entry 2, Table 2), which is virtually irrelevant to cocatalyst feeding, suggestive of the conformation stability of active species. It should be pointed out that this catalytic system showed extremely high activity (8.16 × 10^5^ g (mol of Co)^−1^(h)^−1^), which is comparable with the recent work on rigid Co(II) chloride catalysts (1.37 × 10^5^ g (mol of Co)^−1^(h)^−1^) [68] and α-diimine cobalt catalysts (0.54 × 10^5^ g (mol of Co)^−1^(h)^−1^) [58]. All resultant polyisoprenes are predominantly *cis*-1,4 enchained with about 30% of 3,4 units. Interestingly, the content of the 3,4-unit polymer slightly increased from 31% to 36% with the change in [Co]/[AlEt_2_Cl] ratio from 1/500 to 1/10 (Figure 2 and Figures S11 and S12). The molecular weight of polyisoprene was found to be dependent on the amount of AlEt_2_Cl. The molecular weight of polyisoprene increased gradually with widened molecular weight distribution as the cocatalyst feeding reduced, which could be explained by the occurrence of transfer reaction. In order to give the best cocatalyst feeding, the polymerization under the [Co]/[AlEt_2_Cl] = 1/50 and [Co]/[AlEt_2_Cl] = 1/10 were stopped within 5 min. This proved that a low amount of AlEt_2_Cl has higher activity (entry 4, Table 2). These may be related to the dialkylated species with a significant amount of AlEt_2_Cl, which makes the Co center difficult to cationize to coordinate isoprene. Excitingly, even if the [Co]/[AlEt_2_Cl] ratio was altered to 1/5, the polymerization could still be carried out, and produced a 38% yield of polymer after 1 h, suggestive of the high catalytic ability of the pyridine–oxime ligated Co catalyst. It is worth mentioning that AlEt_2_Cl dosage is much less than in the previous catalyst systems.

The titled complexes **Co2** and **Co3** with methyl and phenyl group were individually tested under the same polymerization conditions for comparison. The isoprene polymerization catalyzed by **Co2**, which had a methyl group on the 6-position of pyridine ring, also showed full conversion at the [Co]/[AlEt_2_Cl] = 1/50 within 10 min (entry 6, Table 2). However, it needs to be emphasized that the pyridine–oxime ligated iron complex bearing one 6-methyl substituent at pyridine ring showed lower activity because of steric hindrance in the previous work [65]. Zhang’s group also reported that the substituent at the 6-position of the pyridine ring significantly influenced the catalytic performances of the iminopyridine ligated Co complexes. For the aldimine- and ketimine-based cobalt complexes, the introduction of a halogen atom (Br) at 6-position of the pyridine ring afforded polymers in higher yields but with a relatively lower molecular weight than the complexes without substituent. The complexes with CH_3_ group produced polymers in lower yields but with higher molecular weights and narrower molecular weight distributions [54]. This may be because that the N-OH group in this work is small enough to tolerates steric hindrance at 6-position of the pyridine ring. When the [Co]/[AlEt_2_Cl] ratio was changed to 1/10, the polymer yield and *cis*-1,4 motif of polyisoprene decreased slightly (entry 7, Table 2). The Ph-substituted complex **Co3** produced polyisoprene with a lower yield at the [Co]/[AlEt_2_Cl] = 1/50. But, similar to complex **Co1**, it exhibited higher reaction activity following the reduction in the amount of AlEt_2_Cl, with [Co]/[AlEt_2_Cl] = 1/10. Though with high hindrance, complexes **Co2** and **Co3** provided polyisoprene paralleling that of **Co1** in terms of microstructure, molecular weight (1.7–2.2 × 10^5^ g/mol) and molecular weight distribution (1.7–2.0). These results indicated that these cobalt catalysts all possess high activity without the effect of steric hindrance.

Furthermore, the catalytic ability of catalyst **Co4** and AlEt_2_Cl is shown in Table 3. Less cocatalyst feeding leads to increased polymer yield, and it displayed the highest activity of 16.3 × 10^5^ g (mol of Co)^−1^(h)^−1^ with [Co]/[AlEt_2_Cl] = 1/50 (entry 2, Table 3). It was interesting that the complex **Co4** exhibited higher activity than **Co1** in terms of the reduction of cocatalyst feeding. However, the polymerization did not conduct at [Co]/[AlEt_2_Cl] = 1/5, even though the reaction time was prolonged to 120 min, which was inferior compared to catalyst **Co1**. The produced polyisoprene possessed a rather balanced molecular weight of 10.2–14.5 × 10^4^ g/mol with unimodal molecular weight distributions. In addition, changing the cocatalyst feeding does not vary the polyisoprene structures with 65% of *cis*-1,4- unit and 35% of 3,4- unit.

## 4. Conclusions

In conclusion, cobalt catalysts bearing pyridine–oxime and picolinaldehyde O-methyl oxime ligands were synthesized and characterized. In combination with AlEt_2_Cl, the Co complexes exhibited a remarkable activity of 1.6 × 10^6^ g (mol of Co)^−1^(h)^−1^ and required only a small amount of cocatalyst feeding ([Co]/[AlEt_2_Cl] = 1/10), which is very promising for the synthetic rubber industry. The complex **Co1**–**Co3** exhibited comparative activity and selectivity to the catalyst **Co4**, and the reaction catalyzed by **Co1** could conduct even the Al feeding as low as to 5. Conversely, the polymerization displayed low conversion at the elevating and reducing reaction temperature, indicating the thermal instability of active species. And, all the catalyst systems provided polyisoprene with parallel molecular weights, molecular weight distribution and *cis*-14- enriched structure.

## Figures and Tables

**Table 1:** Isoprene polymerization using **Co1** and **Co4** with different temperature ^a^.

Entry	Cat.	Temp. (°C)	Yield ^b^ (%)	Act. ^c^ (×10^−5^)	*M_n_*^d^ (×10^−4^)	*M_w_*/*M_n_*^d^	Microstructure(%) ^e^		
							*trans*-1,4	*cis*-1,4	3,4
1	**Co1**	25	95	7.7	12.2	1.6	0	69	31
2	**Co4**	25	83	6.8	10.2	1.7	0	68	32
3	**Co1**	70	83	6.8	17.8	2.0	0	64	36
4	**Co4**	70	56	4.6	4.9	2.0	1	66	33
5	**Co1**	−30	-	-	-	-	-	-	-
6	**Co4**	−30	-	-	-	-	-	-	-

^a^ General conditions: total volume = 8 mL; [isoprene] = 2.5 mol/L; [Co]/[isoprene]/[AlEt_2_Cl] = 1/2000/500; reaction time = 10 min. ^b^ Isolated yield. ^c^ g_Polymer_ (mol of Co)^−1^(h)^−1^. ^d^ Determined by gel permeation chromatography (GPC). ^e^ Determined by ^1^H and ^13^C NMR.

**Table 2:** Isoprene polymerization using **Co1**–**Co3** with AlEt_2_Cl as cocatalyst ^a^.

Entry	Cat.	[Co]/[AlEt_2_Cl]	Time (min)	Yield ^b^ (%)	Act. ^c^ (×10^−5^)	*M*_n_ ^d^ (×10^−4^)	*M_w_*/*M_n_* ^d^	Microstructure(%) ^e^	
								*cis*-1,4	3,4
1	**Co1**	1/50	10	>99	8.2	15.9	1.7	66	34
2	**Co1**	1/10	10	>99	8.2	17.9	1.9	64	36
3	**Co1**	1/50	5	71	11.6	12.8	1.9	66	34
4	**Co1**	1/10	5	83	13.5	10.1	2.1	64	36
5	**Co1**	1/5	60	38	0.5	17.5	1.7	63	37
6	**Co2**	1/50	10	>99	8.2	16.7	1.8	66	34
7	**Co2**	1/10	10	95	7.8	22.0	1.7	64	36
8	**Co3**	1/50	10	89	7.3	17.1	2.0	67	33
9	**Co3**	1/10	10	>99	8.2	18.4	2.0	64	36

^a^ General conditions: total volume = 8 mL; [isoprene] = 2.5 mol/L; [Co]/[isoprene] = 1/2000; 25 °C. ^b^ Isolated yield. ^c^ g_Polymer_ (mol of Co)^−1^(h)^−1^. ^d^ Determined by gel permeation chromatography (GPC). ^e^ Determined by ^1^H and ^13^C NMR.

**Table 3:** Isoprene polymerization using **Co4** with AlEt_2_Cl as cocatalyst ^a^.

Entry	[Co]/[AlEt_2_Cl]	Time (min)	Yield ^b^ (%)	Act. ^c^ (×10^−5^)	*M*_n_ ^d^ (×10^−4^)	*M_w_*/*M_n_* ^d^	Microstructure (%) ^e^	
							*cis*-1,4	3,4
1	1/100	10	93	7.6	10.2	1.7	65	35
2	1/50	5	>99	16.3	14.5	1.8	65	35
3	1/10	5	97	15.8	12.3	2.0	63	37
4	1/5	120	0	-	-	-	-	-

^a^ General conditions: total volume = 8 mL; [isoprene] = 2.5 mol/L; [Co]/[isoprene] = 1/2000; 25 °C. ^b^ Isolated yield. ^c^ g_Polymer_ (mol of Co)^−1^h^−1^. ^d^ Determined by gel permeation chromatography (GPC). ^e^ Determined by ^1^H and ^13^C NMR.

## Data Availability

Data are contained within the article and supplementary materials.

## Supplementary Materials

The following supporting information can be downloaded at: https://www.mdpi.com/2073-4360/15/24/4660/s1, Figures S1–S32: NMR data for polyisoprene. Figures S33–S48: GPC data for polyisoprene. Table S1: Crystal data and structure refinement for complex **Co2**. Table S2: Bond lengths for complex **Co2**. Table S3: Selected bond angles for complex **Co2**.
