# Supplementary material for: Catalytic Behavior of Cobalt Complexes Bearing Pyridine–Oxime Ligands in Isoprene Polymerization
Source: Polymers (Basel). 2023 Dec 10;15(24):4660. doi: 10.3390/polym15244660 (PMC10871112; doi:10.3390/polym15244660)
Supplement: Supplementary file 1 [file polymers-15-04660-s001.zip › polymers-2744383-supplementary.pdf]

Supplementary Information

Catalytic Behavior of Cobalt Complexes Bearing Pyridine–oxime Ligands in Isoprene Polymerization

Yuanxu Du [1,2,†], Shuo Gao [1,†], Hui Ma [1], Siqi Lu [1], Zhenhua Zhang [1,*] and Mengmeng Zhao [1,*]

[1] School of Chemistry and Chemical Engineering, Linyi University, Linyi 276000, China; 202226100109@lyu.edu.cn (Y.D.); gaoshuo9802@163.com (S.G.); maroumodisi@163.com (H.M.); 17866937745@163.com (S.L.);

[2] College of Agriculture and Forestry, Linyi University, Linyi 276000, China

[*] Correspondence: zhangzhenhua@iccas.ac.cn (Z.Z.); zhaomengmeng@lyu.edu.cn (M.Z.)

[†] These authors contributed equally to this work.

X-ray data for complex **Co2** **2**

NMR data for and polyisoprene **5**

GPC data for polyisoprene **21**

X-ray data for complex Co2

Table S1. Crystal data and structure refinement for complex **Co2**.

**Identification Code**	**211113a**
Empirical formula	$C_{14}H_{16}Cl_2CoN_4O_2$
Formula weight	402.14
Temperature	298(2) K
Wavelength	0.71073 Å
Crystal system, space group	Monoclinic, C2/c
	a = 16.4302(18) Å alpha = 90 deg.
Unit cell dimensions	b = 7.8040(8) Å beta = 114.920(6) deg.
	c = 14.9001(15) Å gamma = 90 deg.
Volume	1732.6(3) A^3
Z, Calculated density	4, 1.542 Mg/m^3
Absorption coefficient	1.311 mm^1
F(000)	820
Crystal size	0.13 × 0.07 × 0.04 mm^3
Theta range for data collection	2.73 to 25.02 deg.
Limiting indices	$-19 \leqslant h \leq 17, -9 \leq k \leq 9, -15 \leq l \leq 17$
Reflections collected / unique	2960/2960 [R(int) = 0.0000]
Completeness to theta = 25.02	97.1%
Absorption correction	Semi-empirical from equivalents
Max. and min. transmission	0.9494 and 0.8480
Refinement method	Full-matrix least-squares on F^2
Data / restraints / parameters	2960 / 608 / 214
Goodness-of-fit on F^2	1.065
Final R indices [I>2sigma(I)]	R1 = 0.1481, wR2 = 0.2869
R indices (all data)	R1 = 0.2954, wR2 = 0.3424
Largest diff. peak and hole	1.258 and -1.730 $e.A^3$

Table S2. Bond lengths for complex **Co2**.

bond lengths (Å)	
Co(1)-N(2)	2.098(16)
Co(1)-N(4)	2.132(16)
Co(1)-N(1)	2.188(17)
Co(1)-N(3)	2.219(16)
Co(1)-Cl(2)	2.455(6)
Co(1)-Cl(1)	2.470(7)
N(1)-C(6)	1.31(2)
N(1)-C(2)	1.37(2)
N(2)-C(1)	1.27(3)
N(2)-O(1)	1.340(19)
N(3)-C(13)	1.35(2)
N(3)-C(9)	1.43(2)
N(4)-C(8)	1.29(2)
N(4)-O(2)	1.39(2)

Table S3. Selected bond angles for complex **Co2**.

**selected bond angles (°)**			
N(2)-Co(1)-N(4)	172.1(5)	C(6)-N(1)-C(2)	113.1(18)
N(2)-Co(1)-N(1)	77.2(6)	C(6)-N(1)-Co(1)	130.9(14)
N(4)-Co(1)-N(1)	109.6(6)	C(2)-N(1)-Co(1)	114.4(13)
N(2)-Co(1)-N(3)	109.5(6)	C(1)-N(2)-O(1)	116.5(16)
N(4)-Co(1)-N(3)	74.7(6)	C(1)-N(2)-Co(1)	114.8(14)
N(1)-Co(1)-N(3)	94.0(4)	O(1)-N(2)-Co(1)	128.1(12)
N(2)-Co(1)-Cl(2)	83.6(5)	C(13)-N(3)-C(9)	117.9(16)
N(4)-Co(1)-Cl(2)	89.9(5)	C(13)-N(3)-Co(1)	130.7(12)
N(1)-Co(1)-Cl(2)	160.2(5)	C(9)-N(3)-Co(1)	107.8(12)
N(3)-Co(1)-Cl(2)	87.7(4)	C(8)-N(4)-O(2)	116.3(15)
N(2)-Co(1)-Cl(1)	90.6(5)	C(8)-N(4)-Co(1)	121.0(15)
N(4)-Co(1)-Cl(1)	85.9(5)	O(2)-N(4)-Co(1)	122.7(11)
N(1)-Co(1)-Cl(1)	86.5(5)	N(2)-O(1)-H(1)	109.5
N(3)-Co(1)-Cl(1)	159.6(5)	N(4)-O(2)-H(2)	109.5
Cl(2)-Co(1)-Cl(1)	98.68(15)	N(2)-C(1)-C(2)	120.9(19)

NMR data for polyisoprene

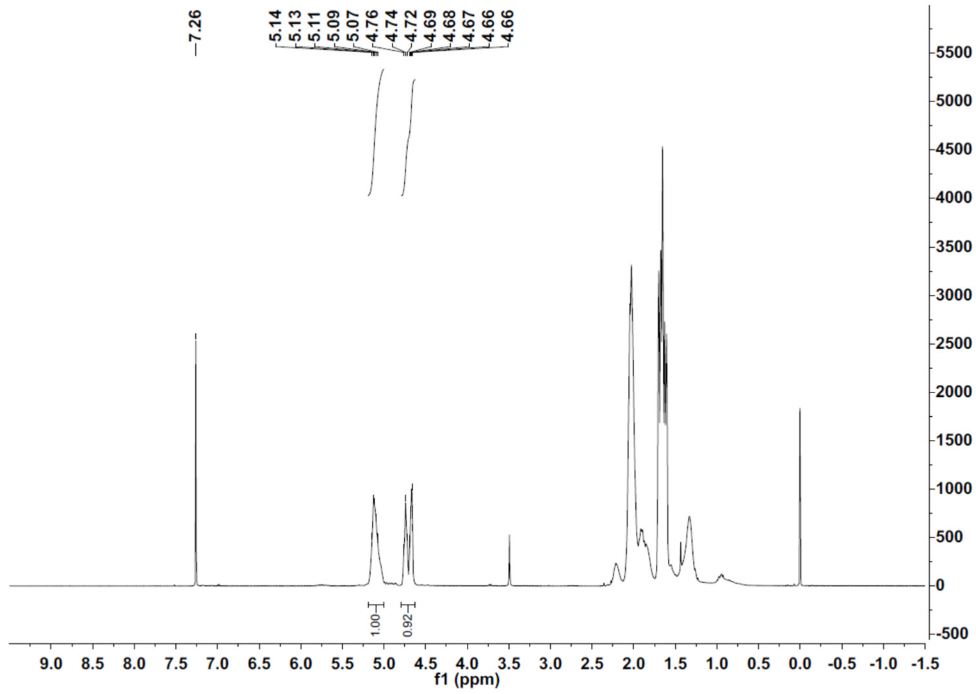

Figure S1. Table 1, entry 1 (^{1}H MMR)

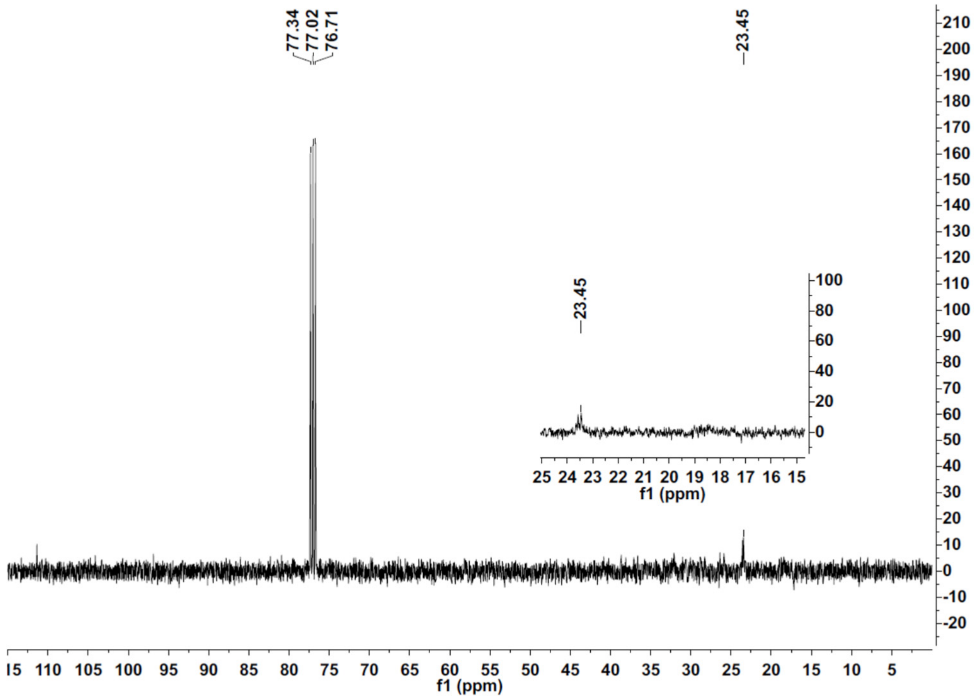

Figure S2. Table 1, entry 1 (^{13}C MMR)

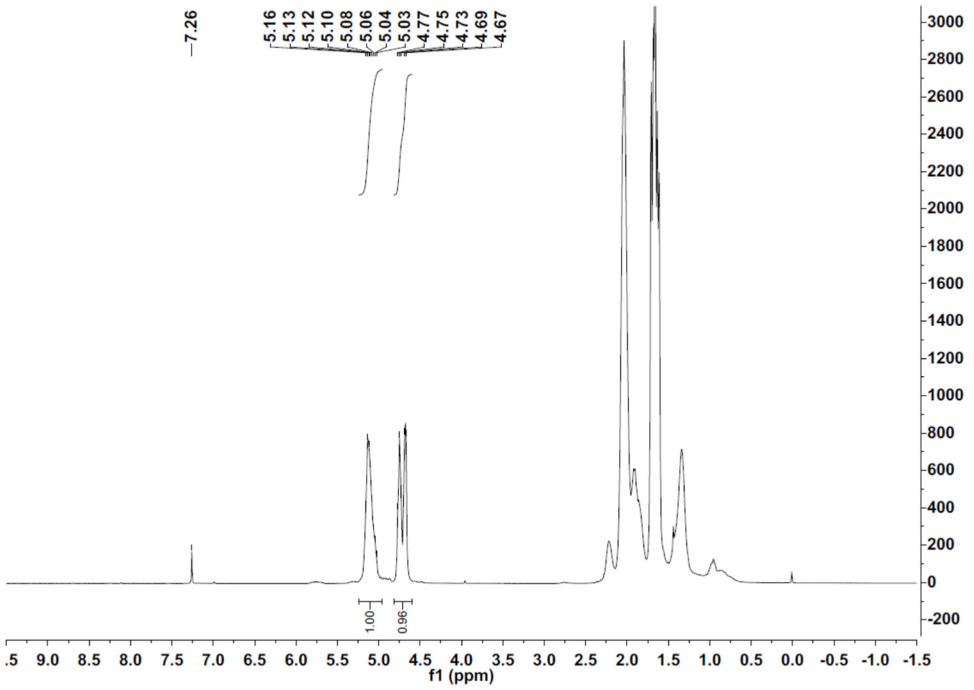

Figure S3. Table 1, entry 2 (^{1}H MMR)

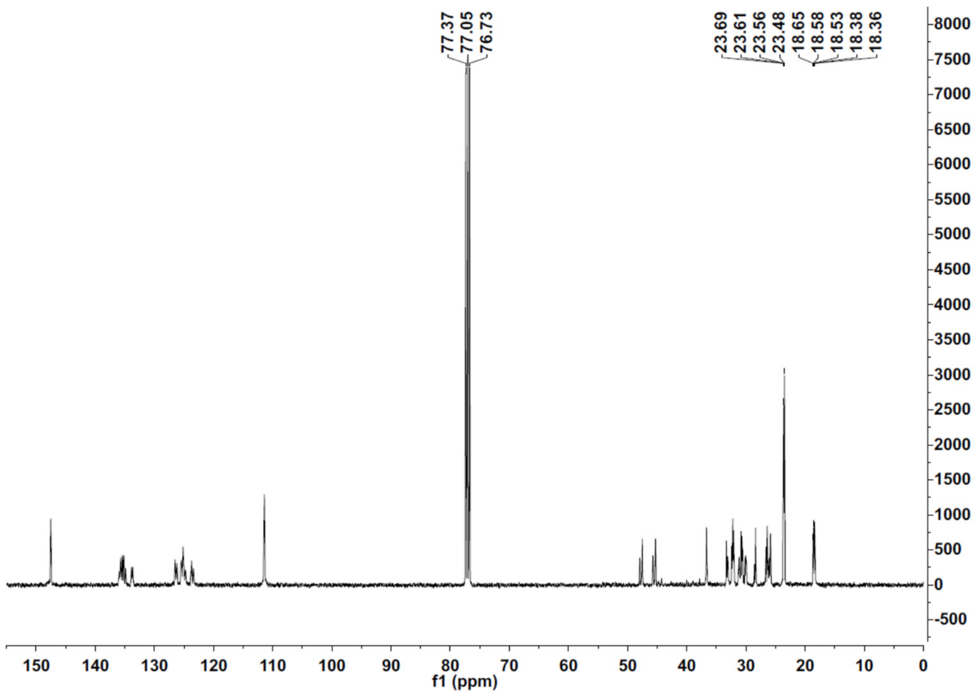

Figure S4. Table 1, entry 2 (^{13}C MMR)

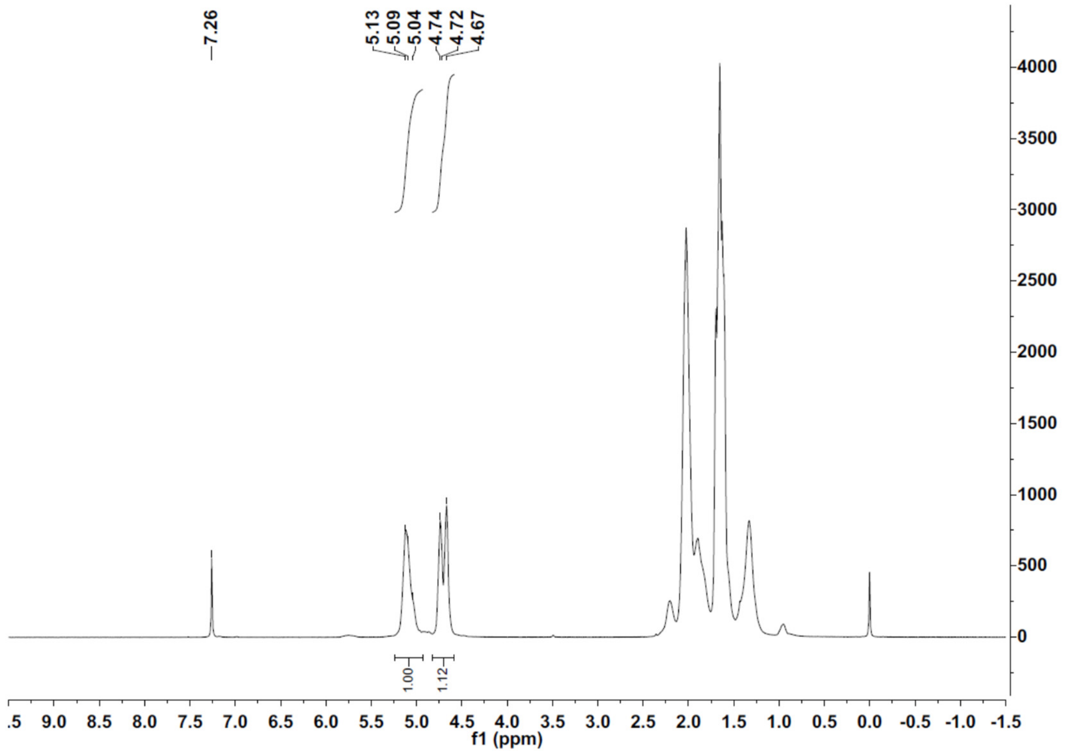

Figure S5. Table 1, entry 3 (^{1}H MMR)

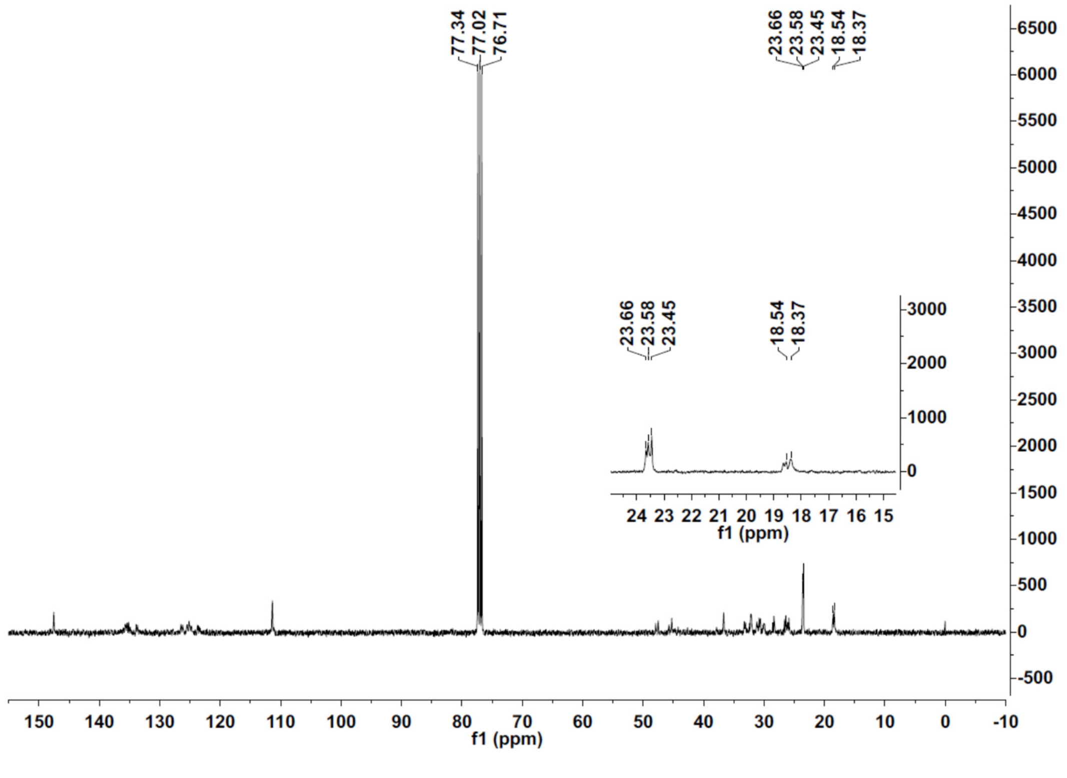

Figure S6. Table 1, entry 3 (^{13}C MMR)

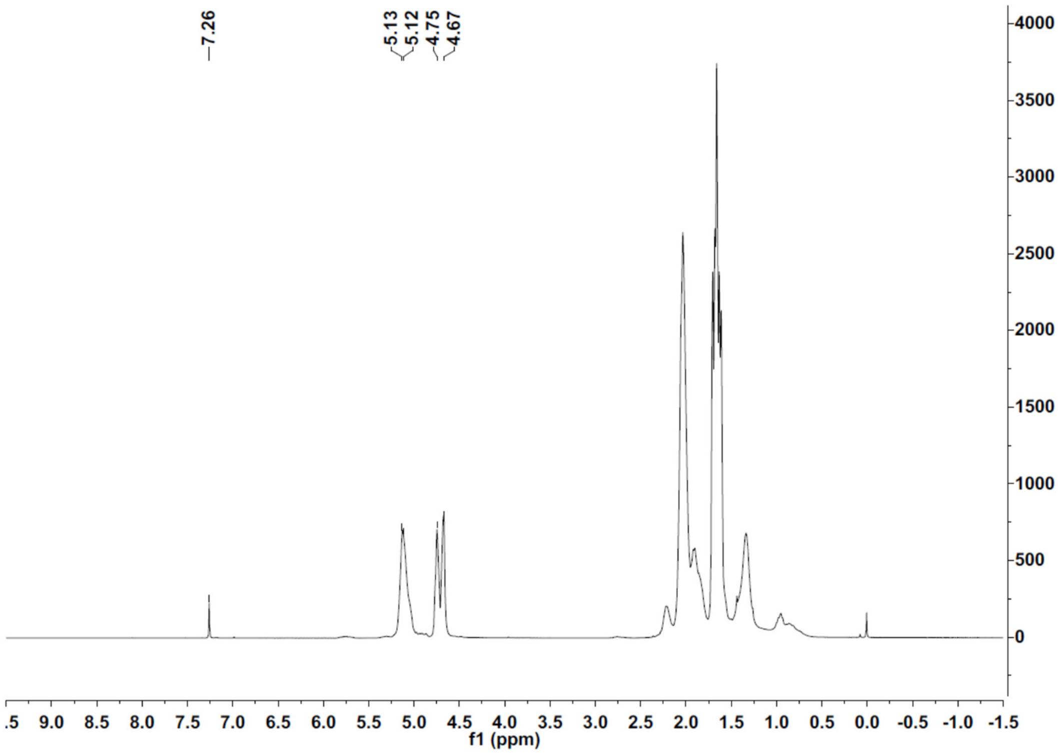

Figure S7. Table 1, entry 4 (^{1}H MMR)

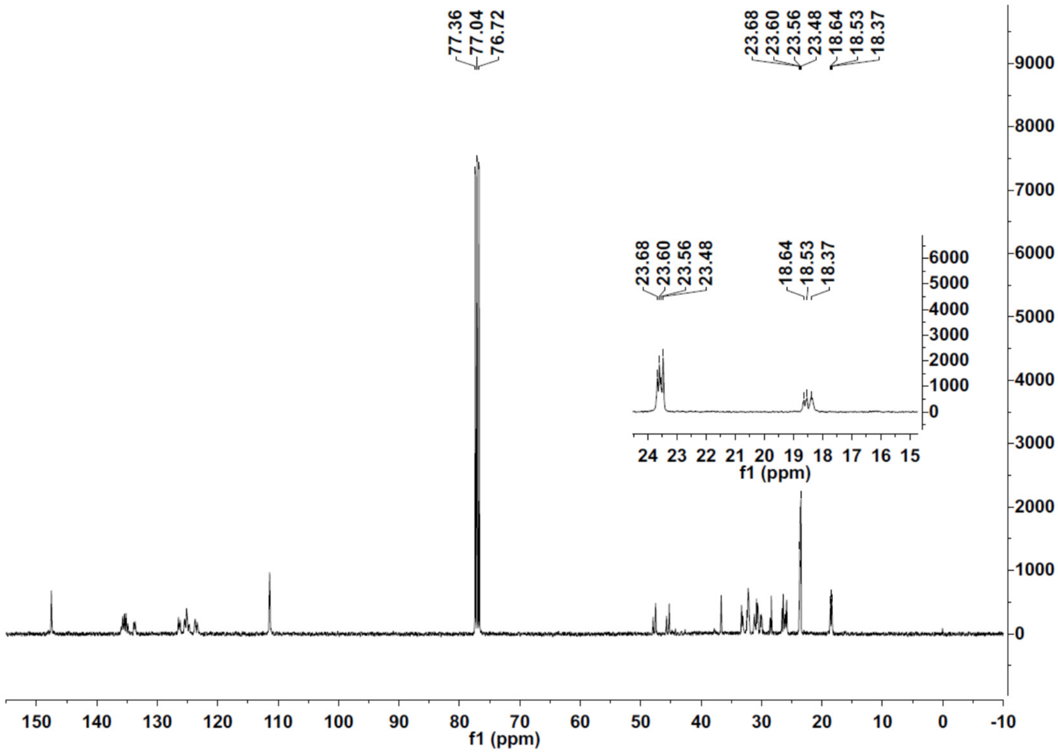

Figure S8. Table 1, entry 4 (^{13}C MMR)

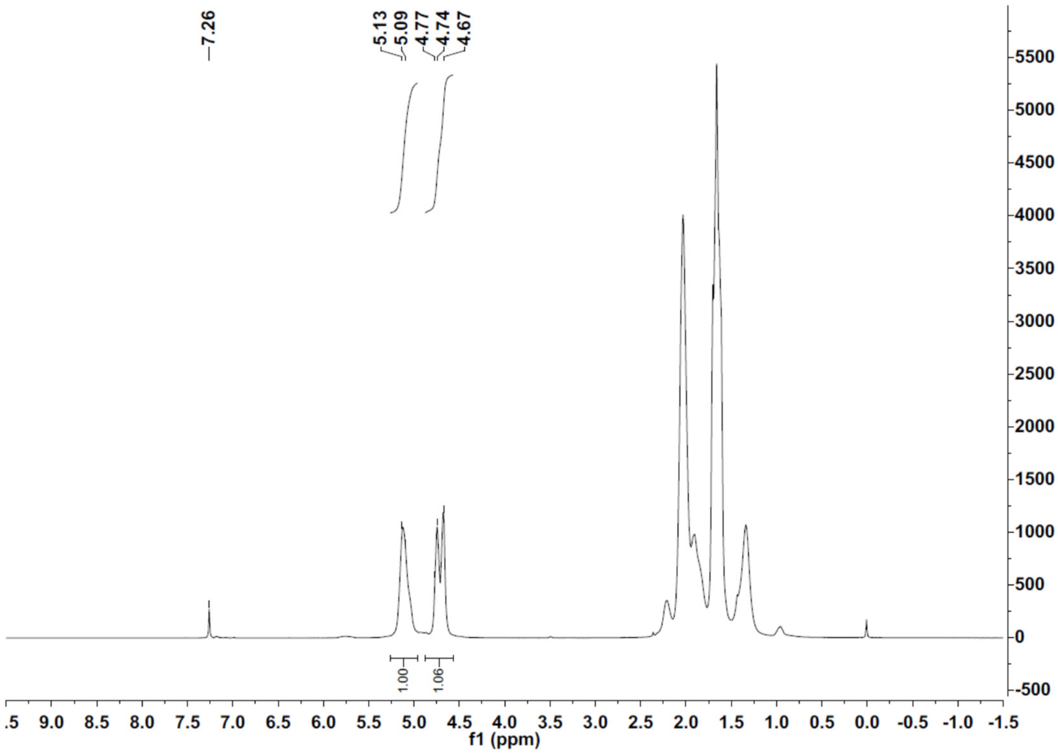

Figure S9. Table 2, entry 1 (^{1}H MMR)

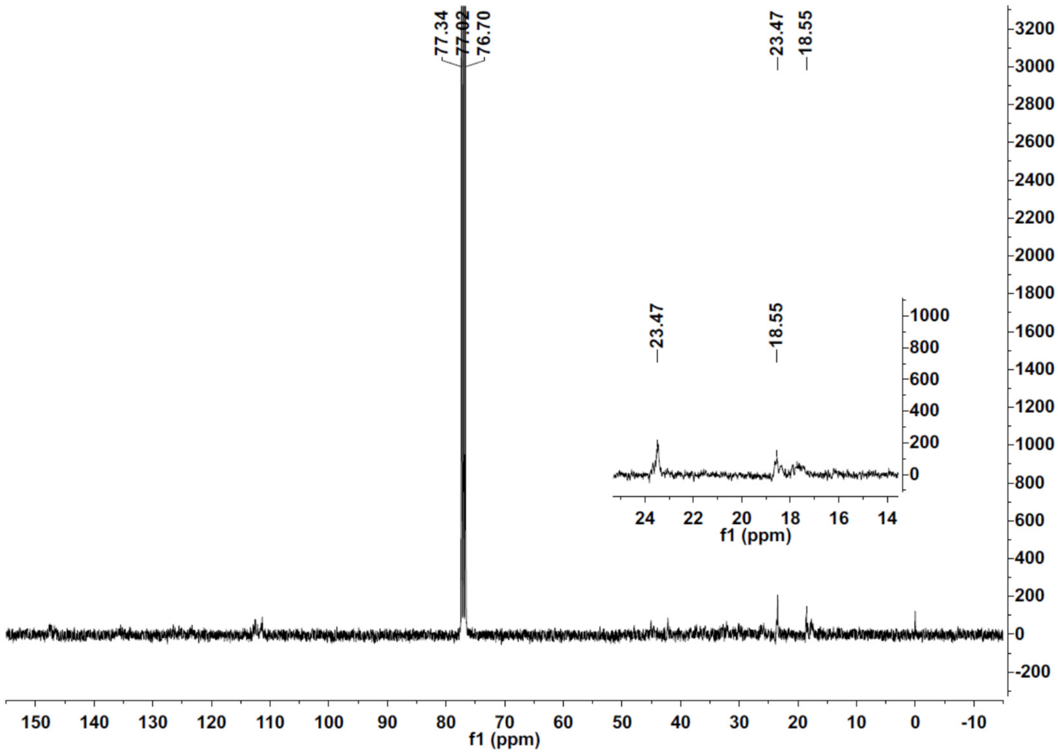

Figure S10. Table 2, entry 1 (^{13}C MMR)

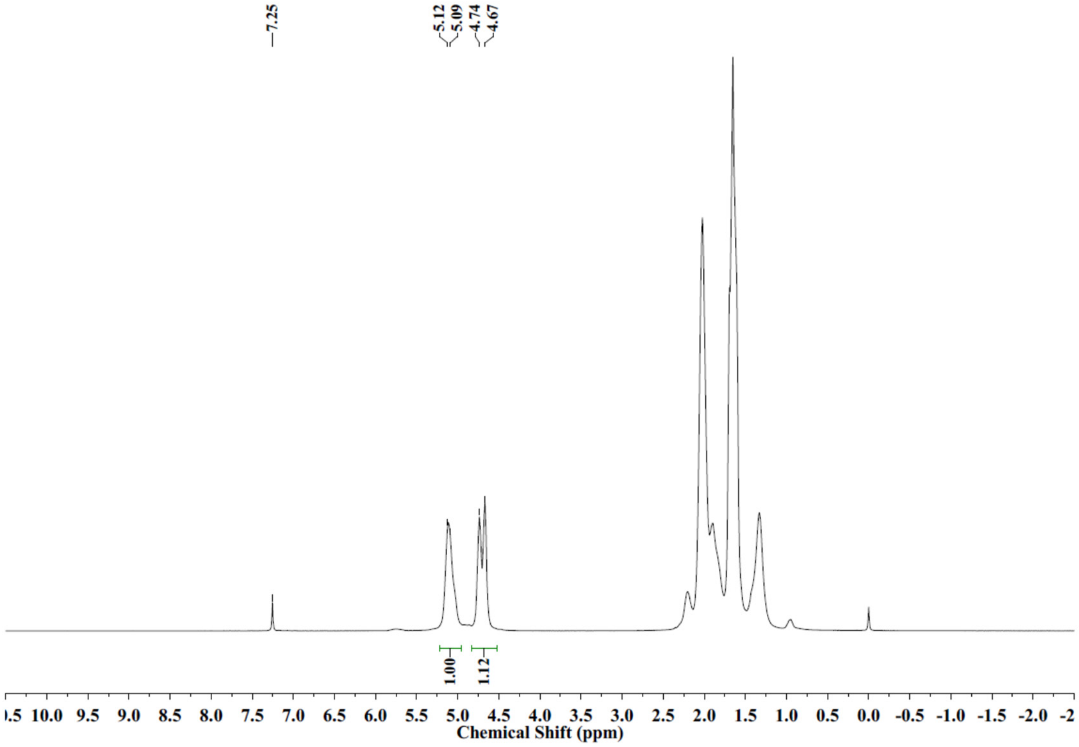

Figure S11. Table 2, entry 2 (^{1}H MMR)

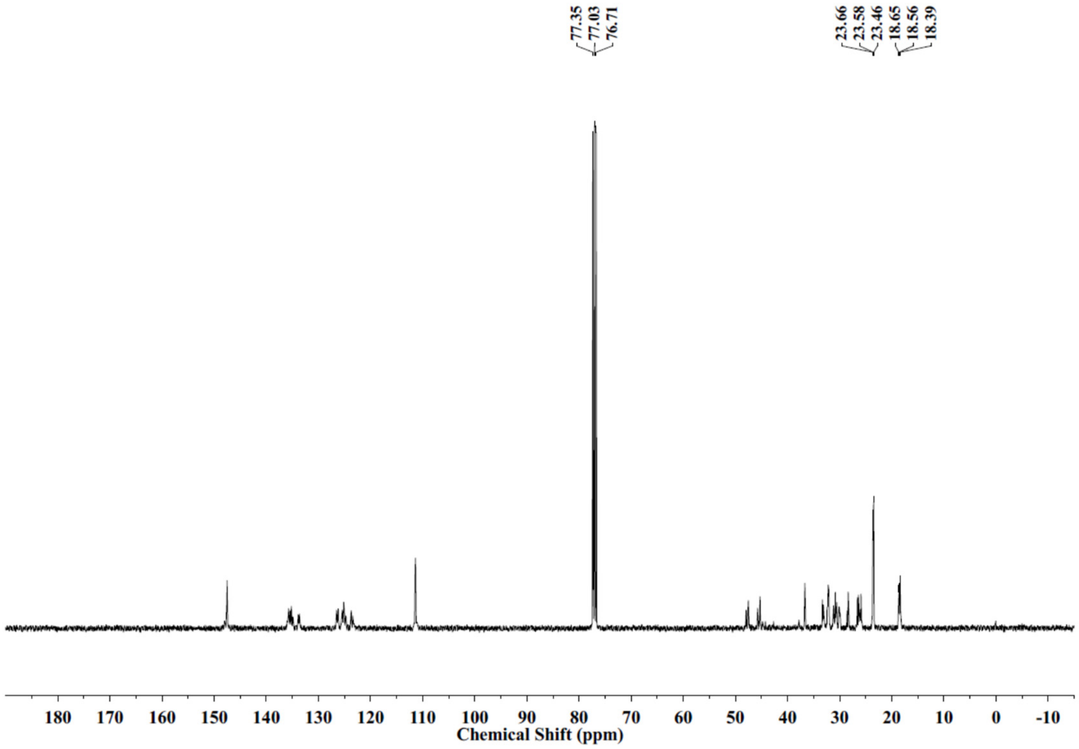

Figure S12. Table 2, entry 2 (^{13}C MMR)

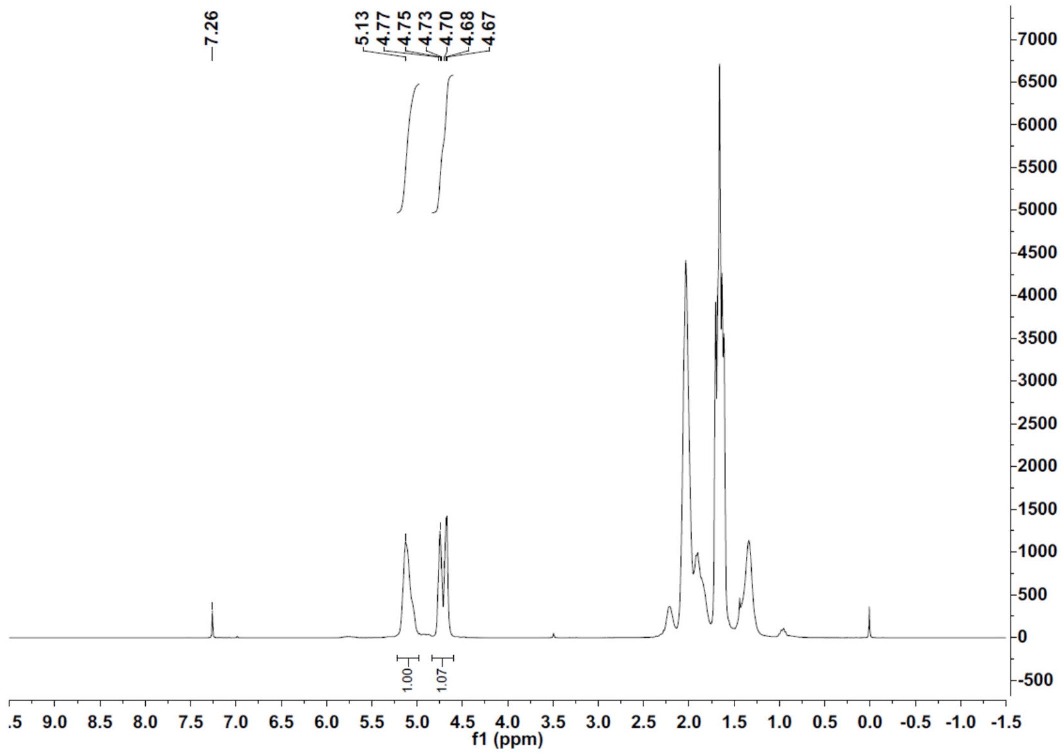

Figure S13. Table 2, entry 3 (^{1}H MMR)

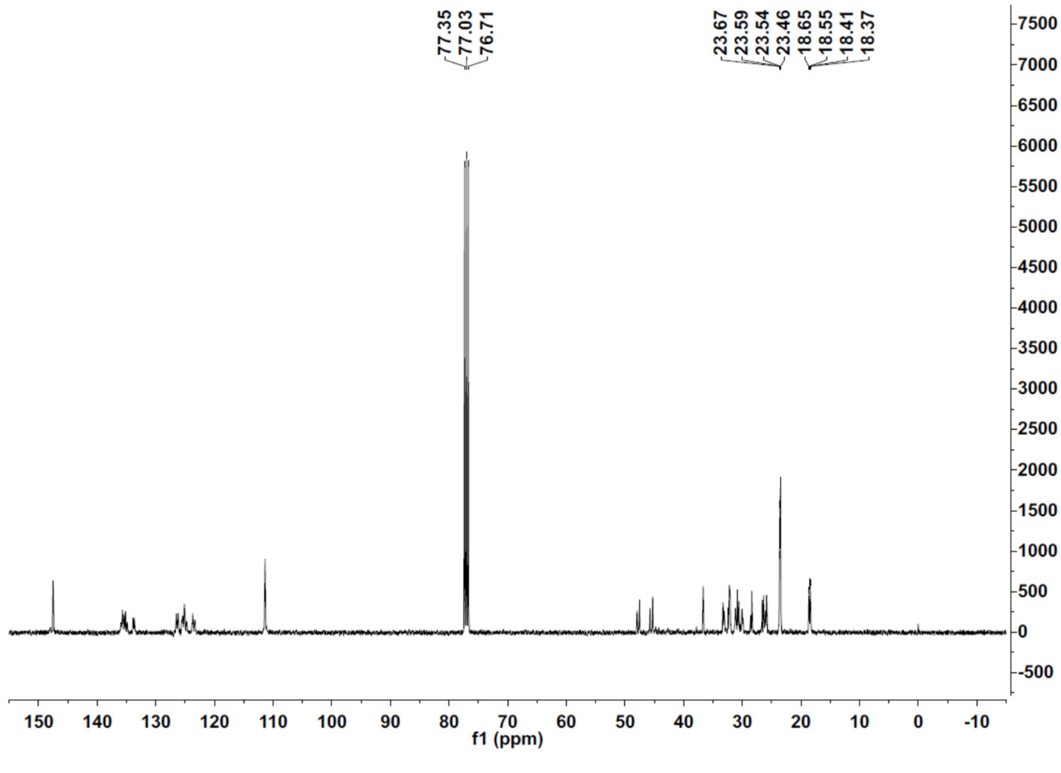

Figure S14. Table 2, entry 3 (^{13}C MMR)

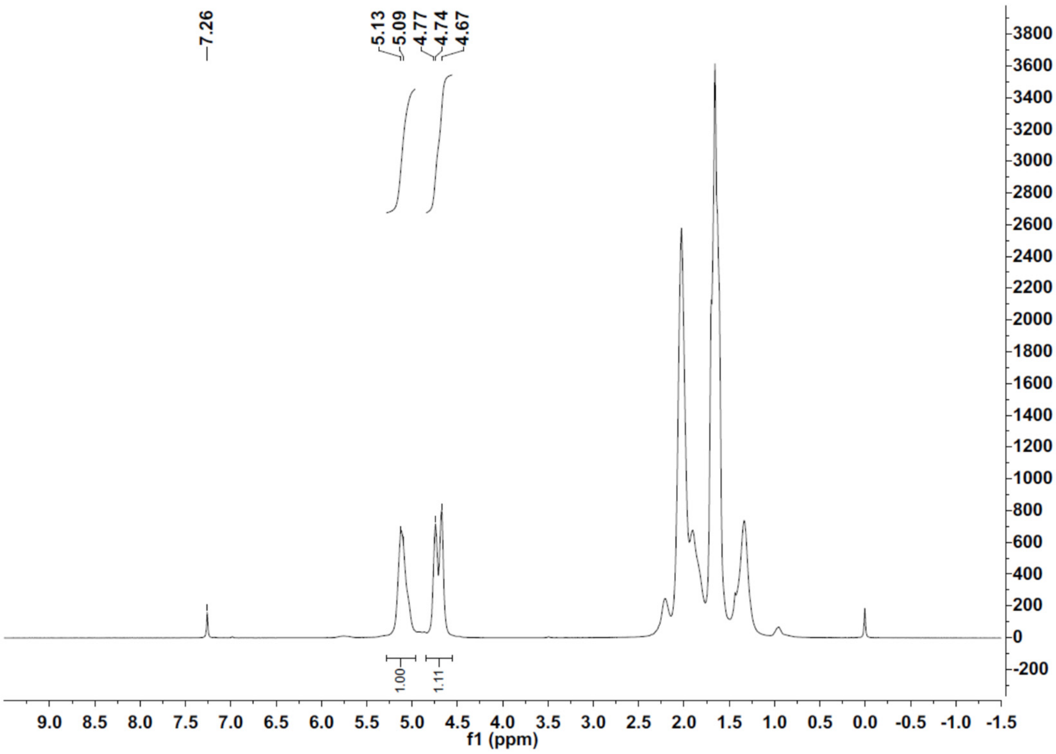

Figure S15. Table 2, entry 4 (^{1}H MMR)

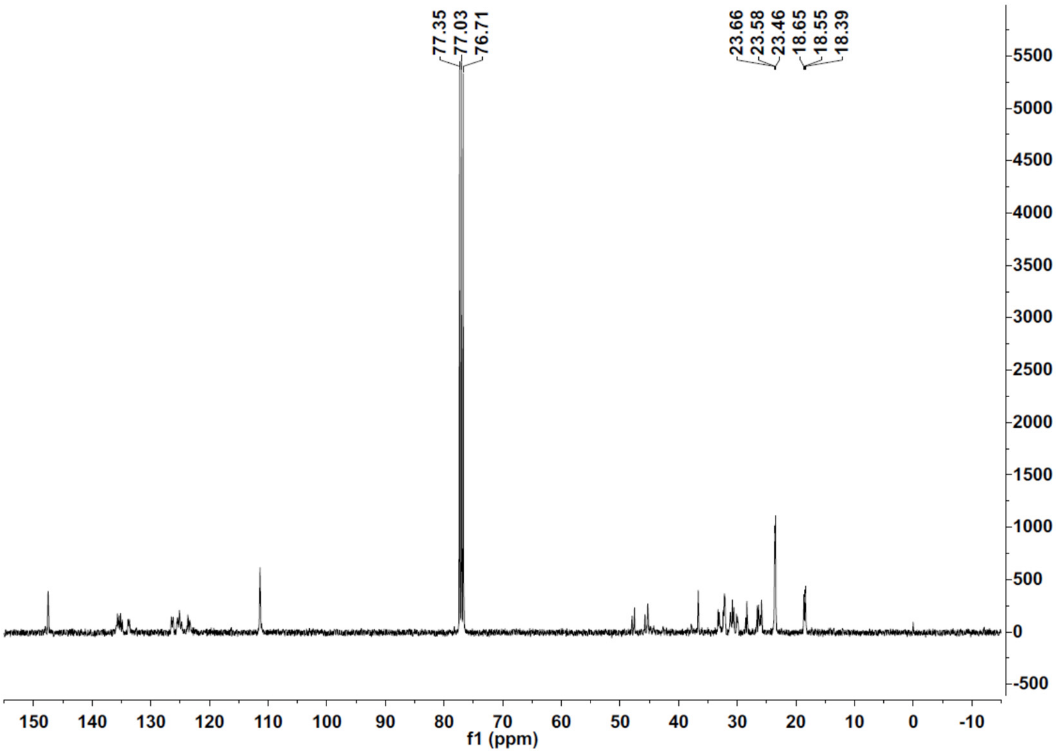

Figure S16. Table 2, entry 4 (^{13}C MMR)

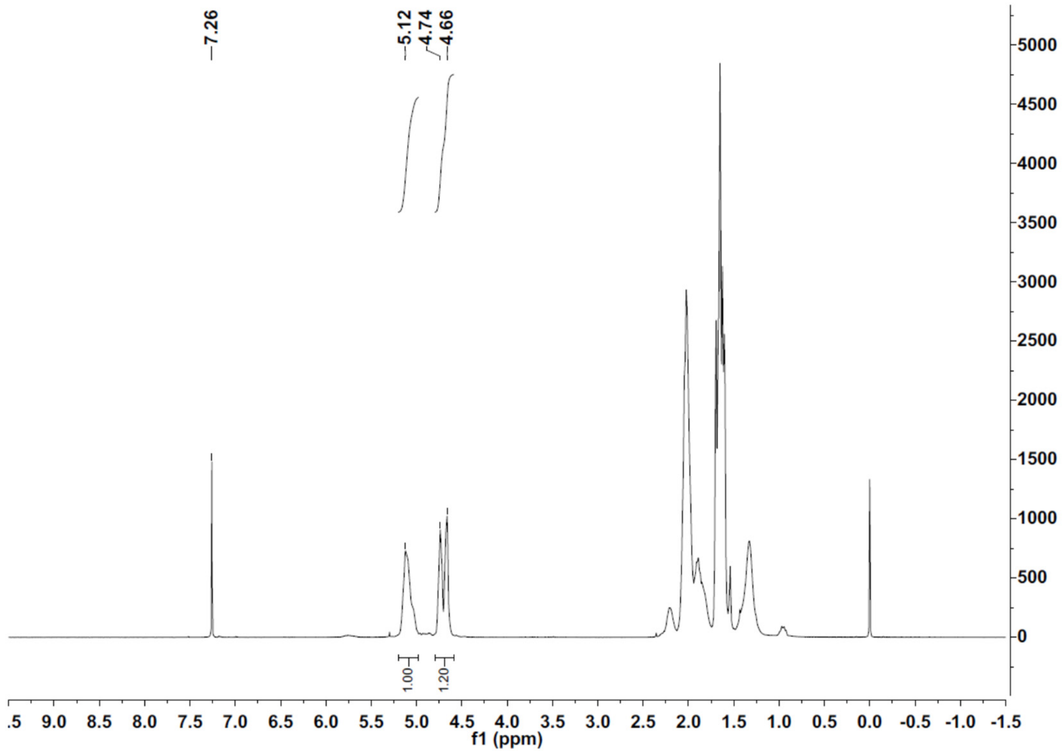

Figure S17. Table 2, entry 5 (^{1}H MMR)

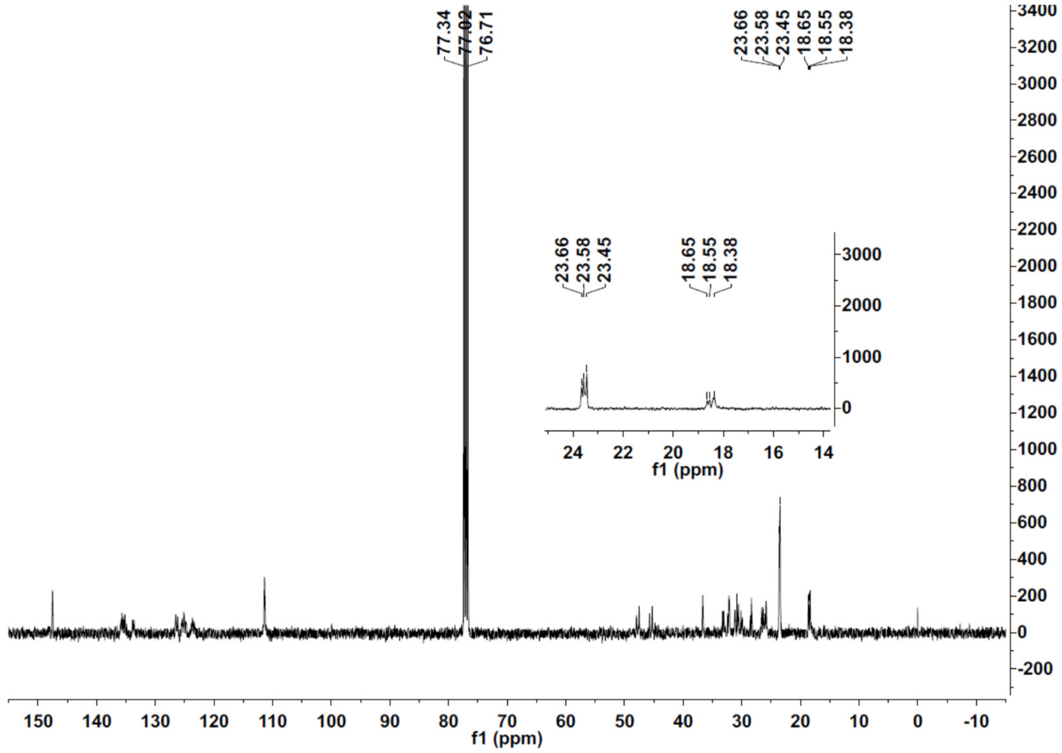

Figure S18. Table 2, entry 5 (^{13}C MMR)

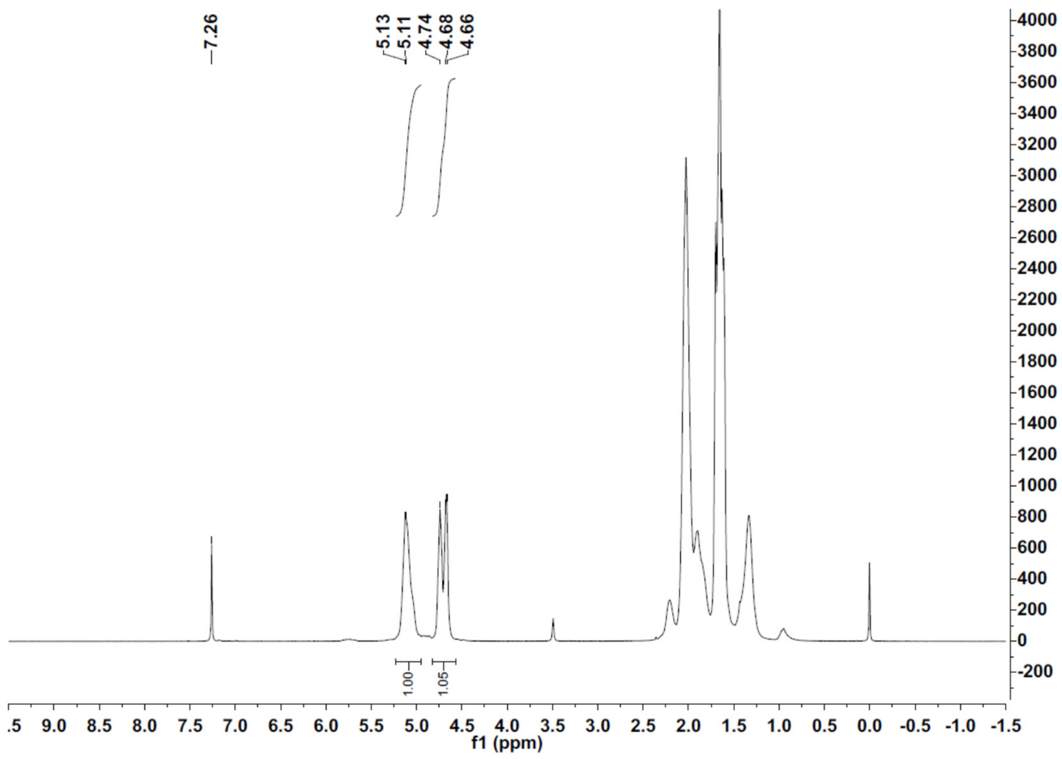

Figure S19. Table 2, entry 6 (^{1}H MMR)

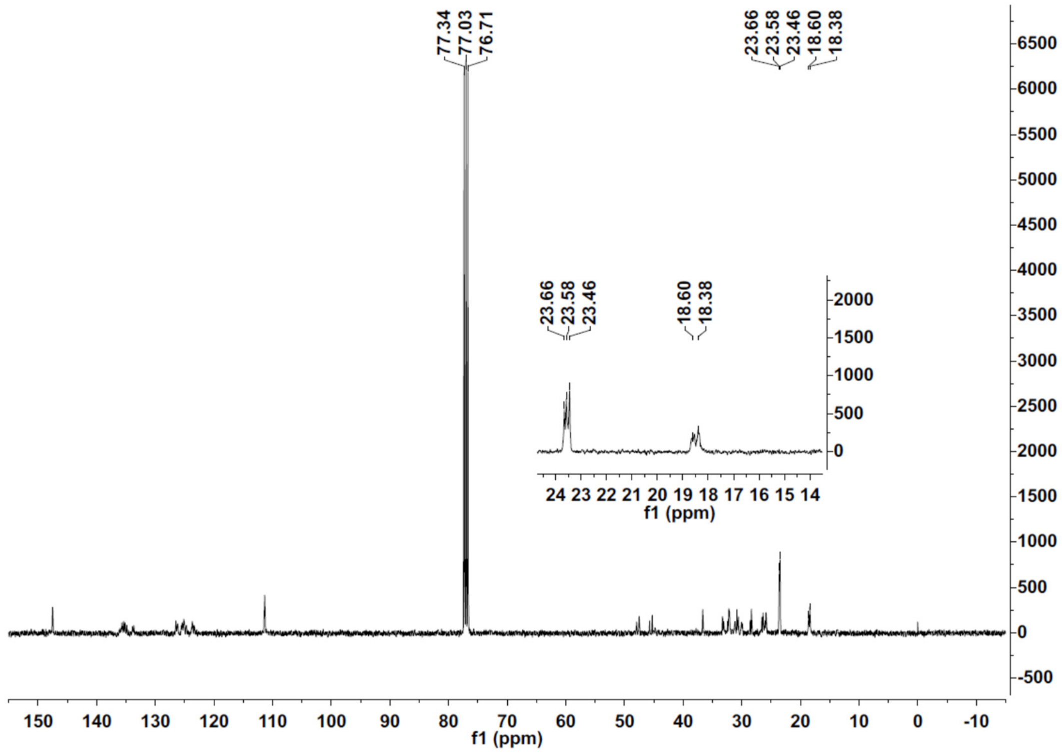

Figure S20. Table 2, entry 6 (^{13}C MMR)

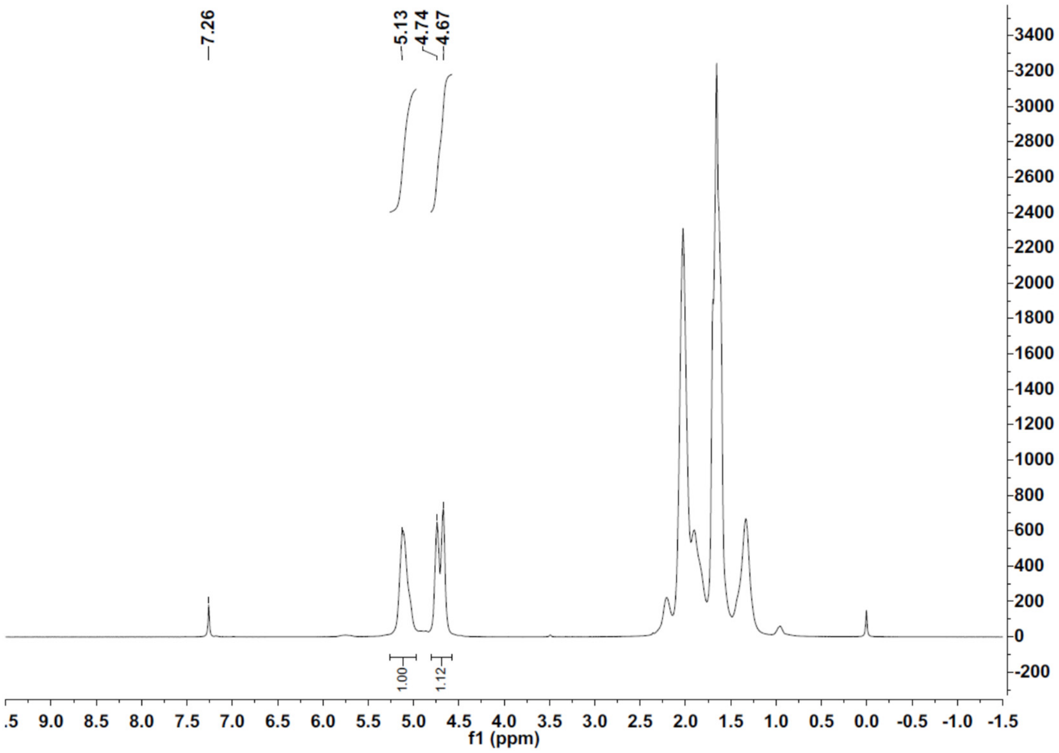

Figure S21. Table 2, entry 7 (^{1}H MMR)

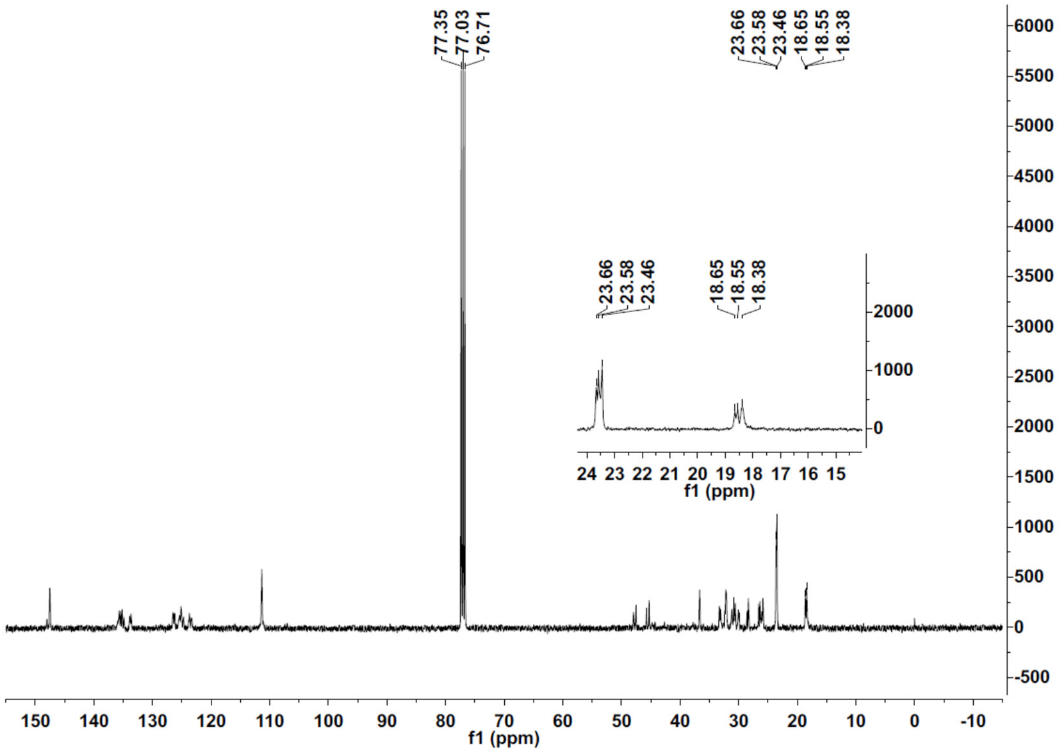

Figure S22. Table 2, entry 7 (^{13}C MMR)

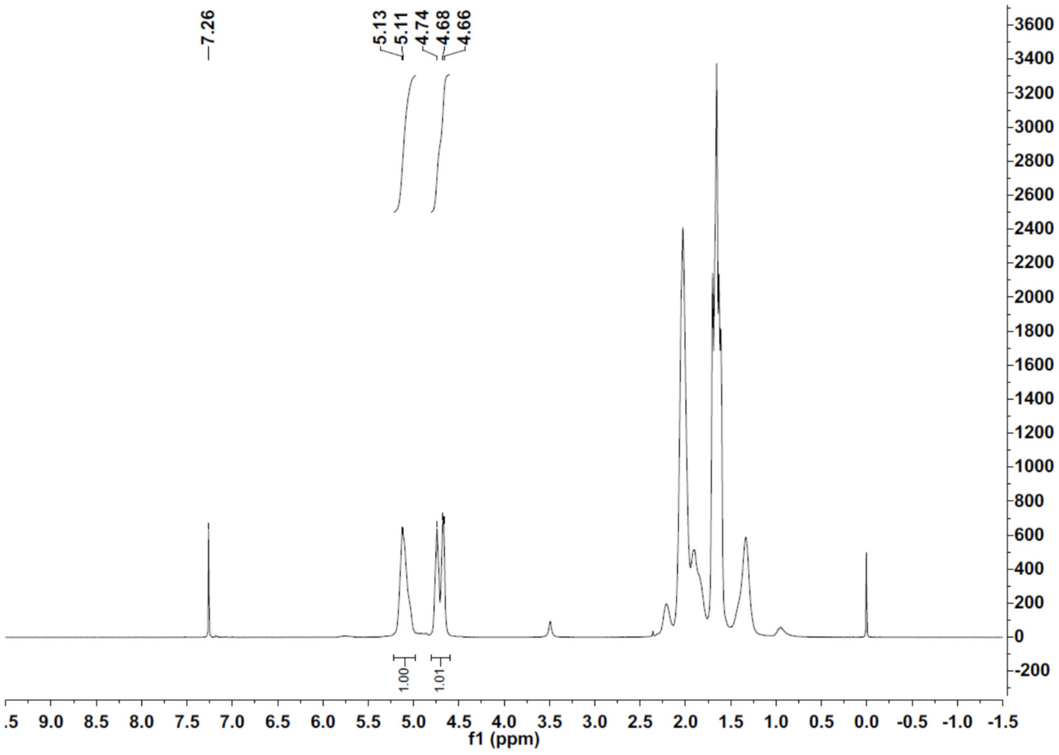

Figure S23. Table 2, entry 8 (^{1}H MMR)

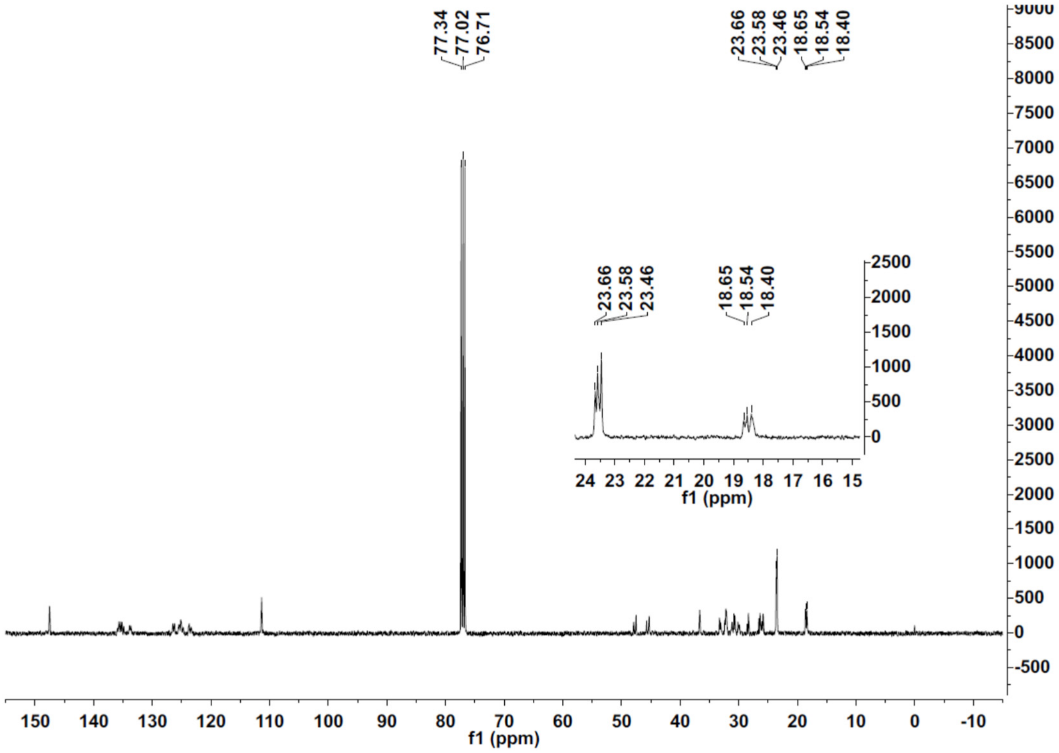

Figure S24. Table 2, entry 8 (^{13}C MMR)

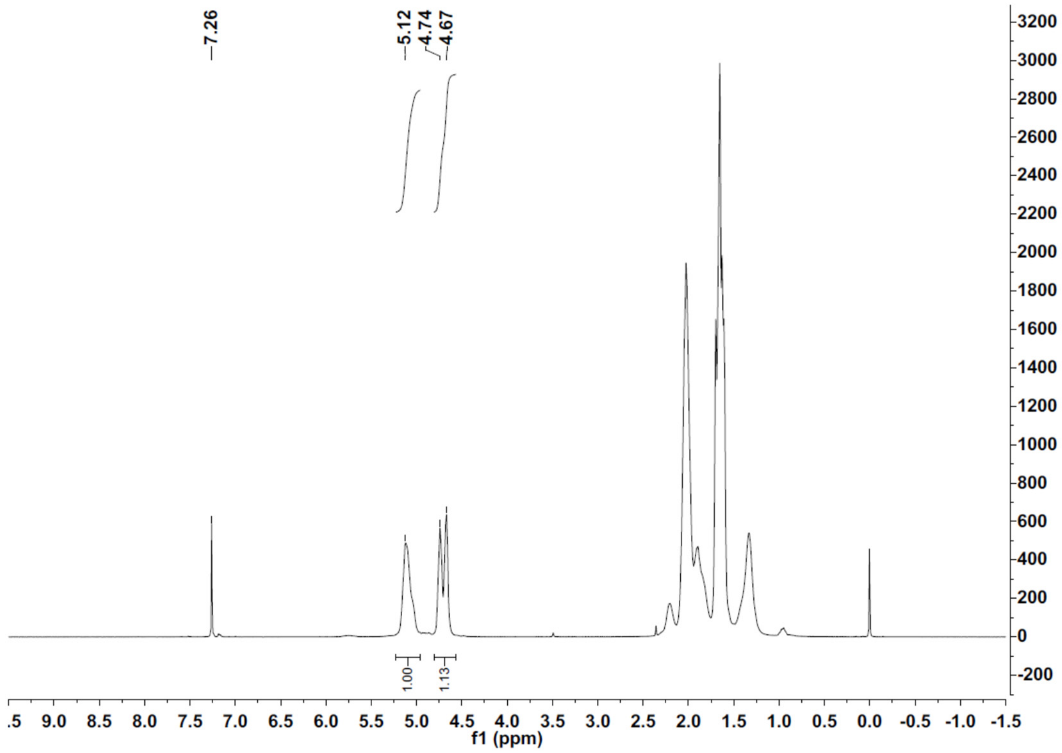

Figure S25. Table 2, entry 9 (^{1}H MMR)

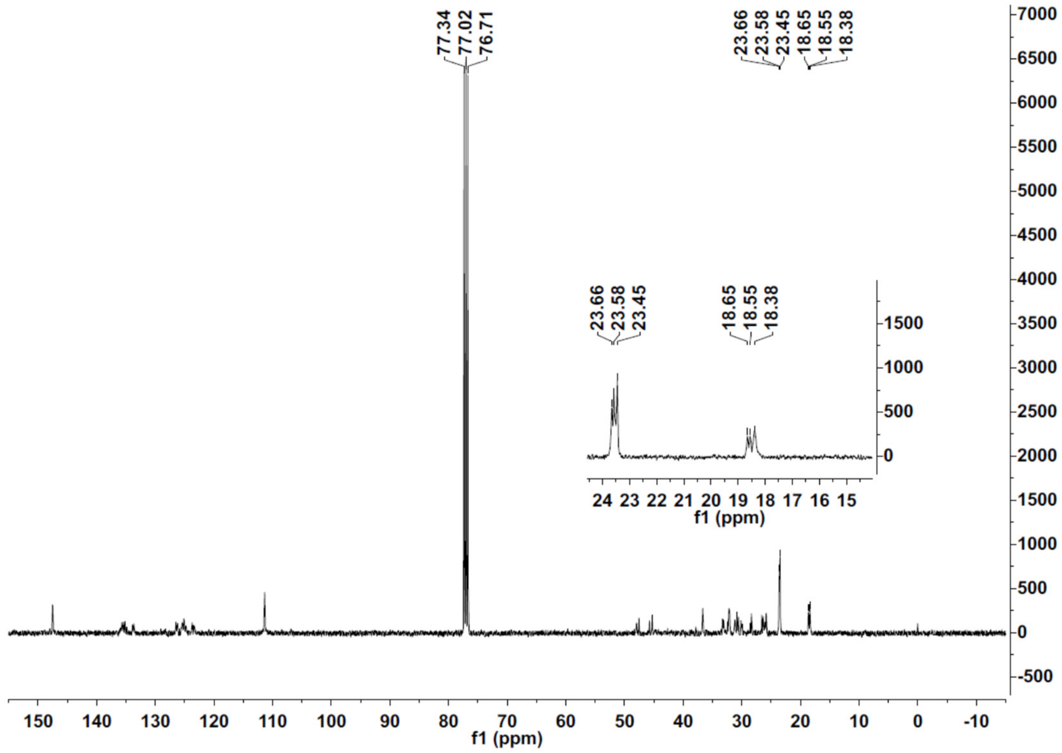

Figure S26. Table 2, entry 9 (^{13}C MMR)

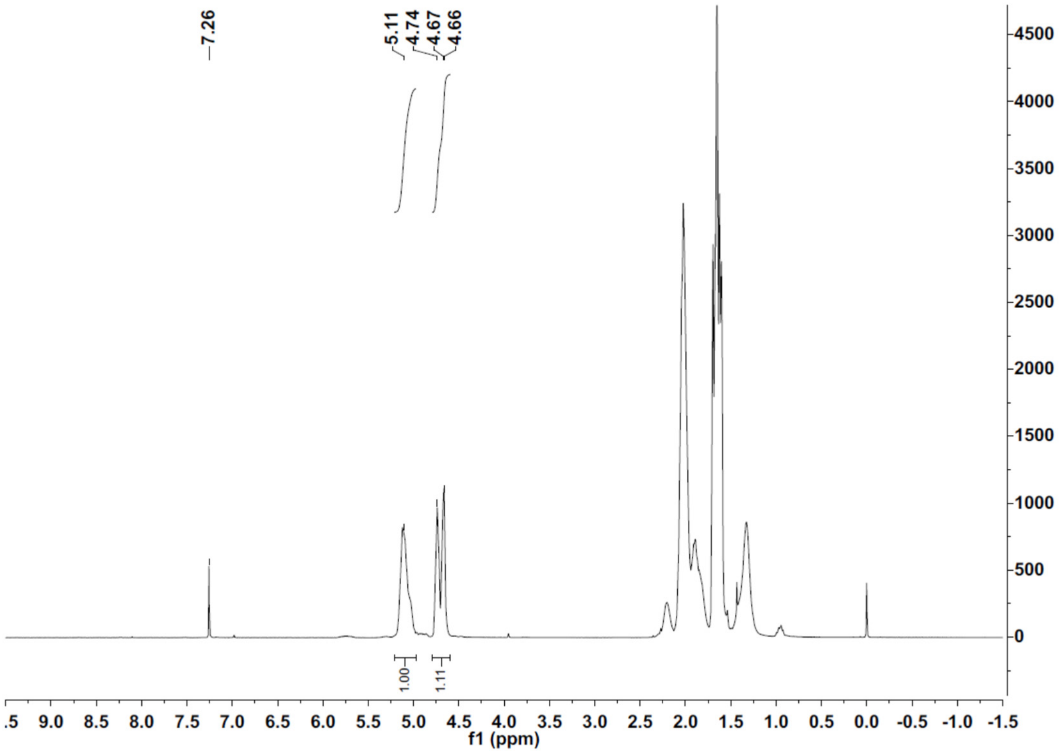

Figure S27. Table 3, entry 1 (^{1}H MMR)

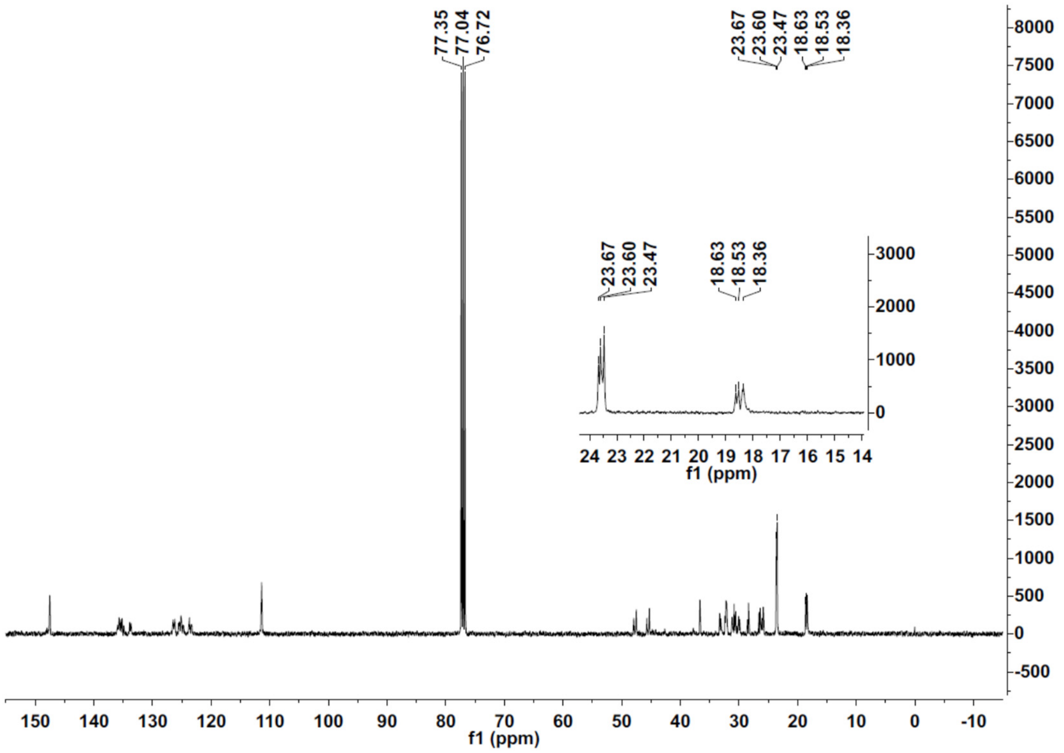

Figure S28. Table 3, entry 1 (^{13}C MMR)

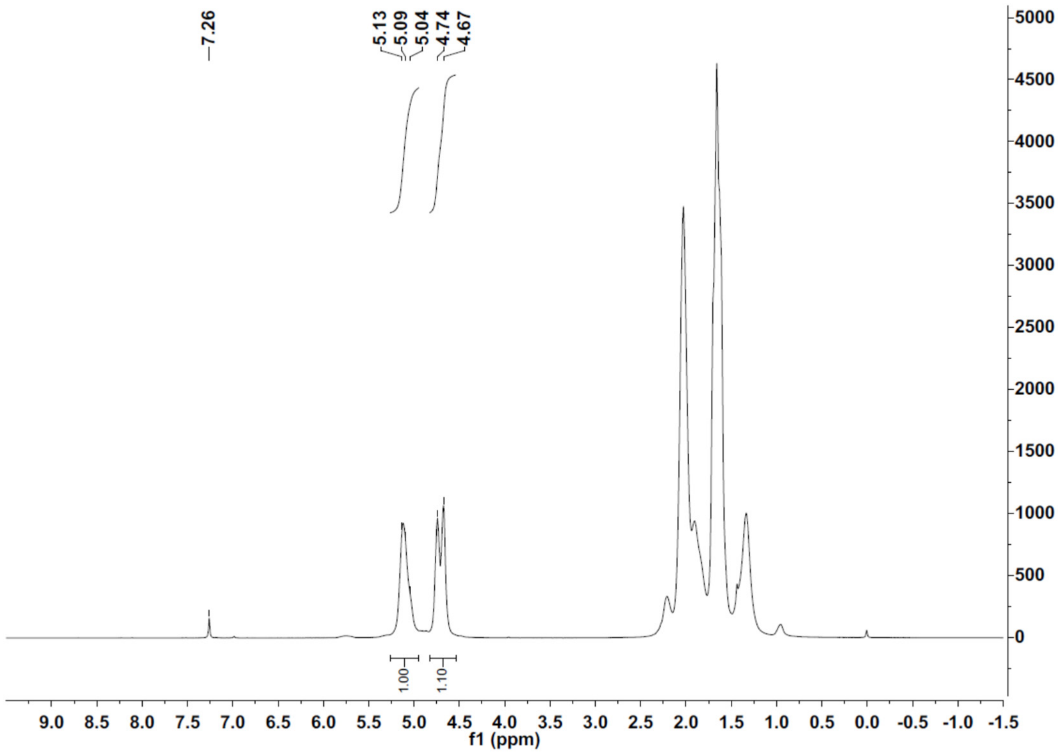

Figure S29. Table 3, entry 2 (^{1}H MMR)

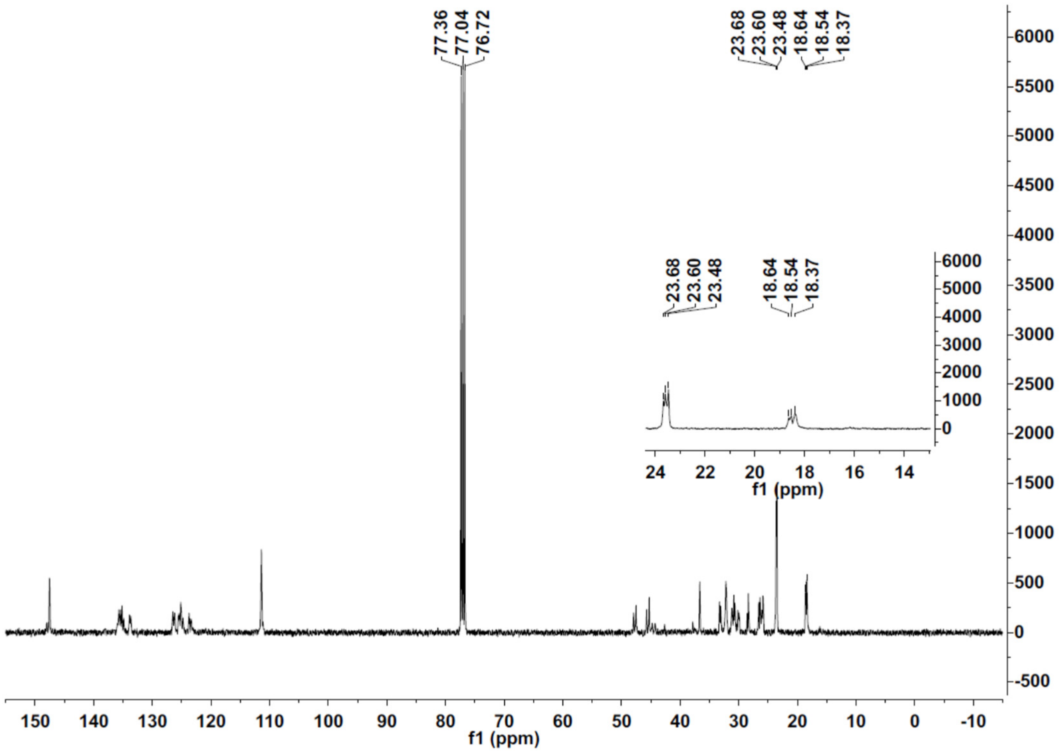

Figure S30. Table 3, entry 2 (^{13}C MMR)

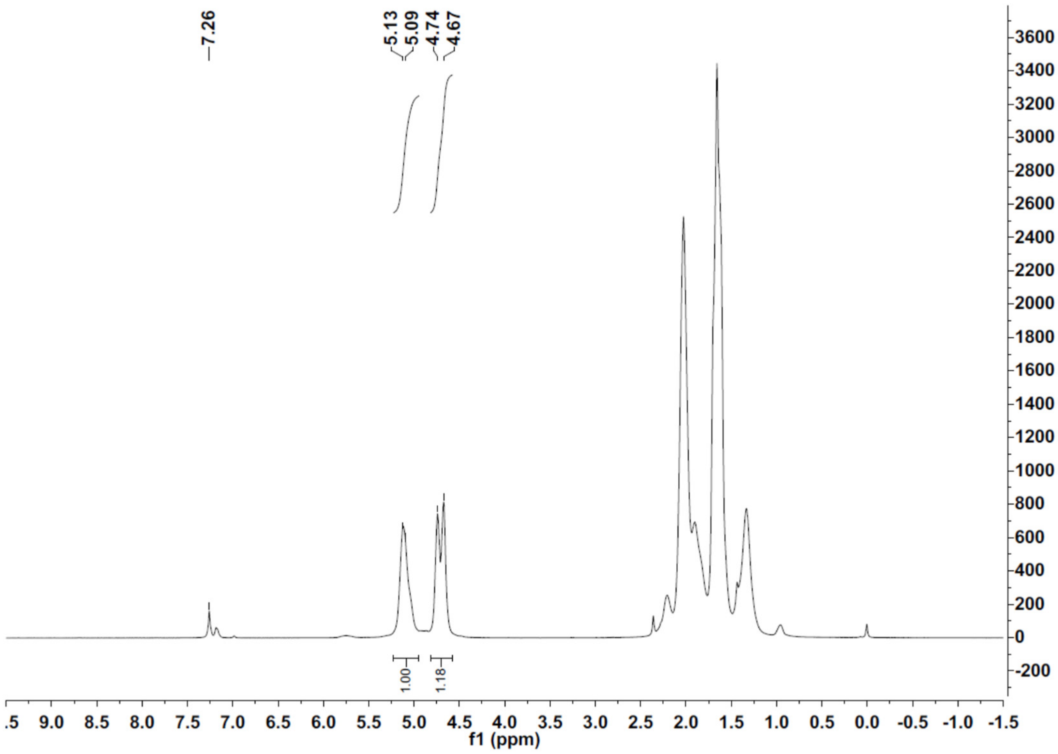

Figure S31. Table 3, entry 3 (¹H MMR)

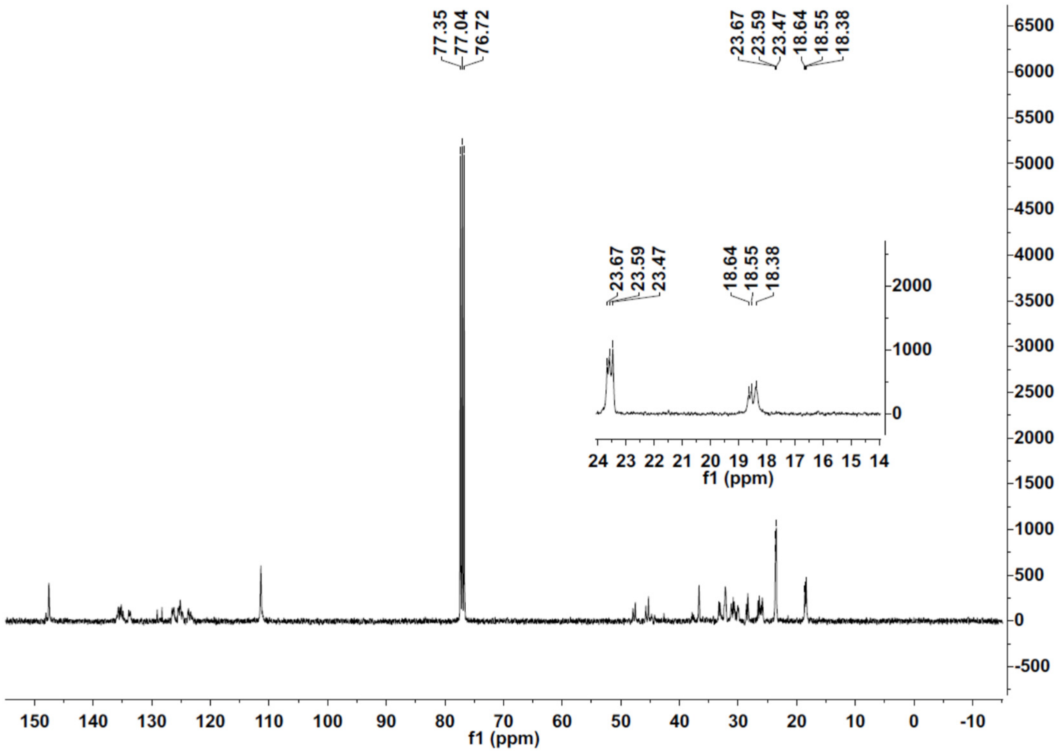

Figure S32. Table 3, entry 3 (¹³C MMR)

GPC data for polyisoprene

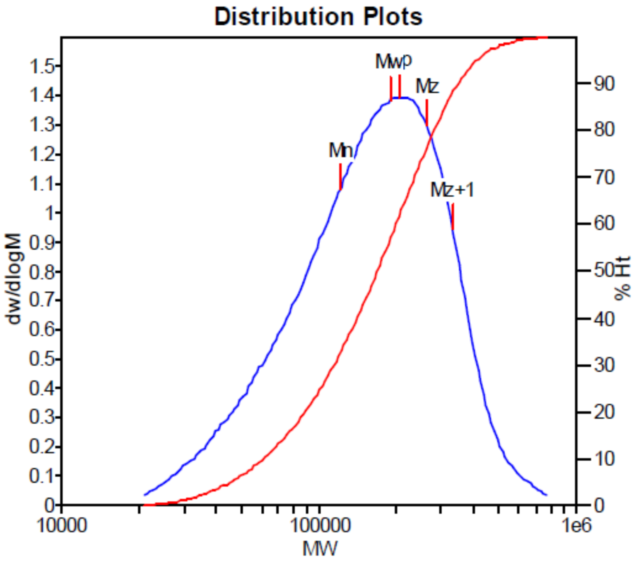

MW Averages

Peak No	Mp	Mn	Mw	Mz	Mz+1	Mv	PD
1	205180	121676	189544	261957	332080	178213	1.55778

Figure S33. Table 1, entry 1

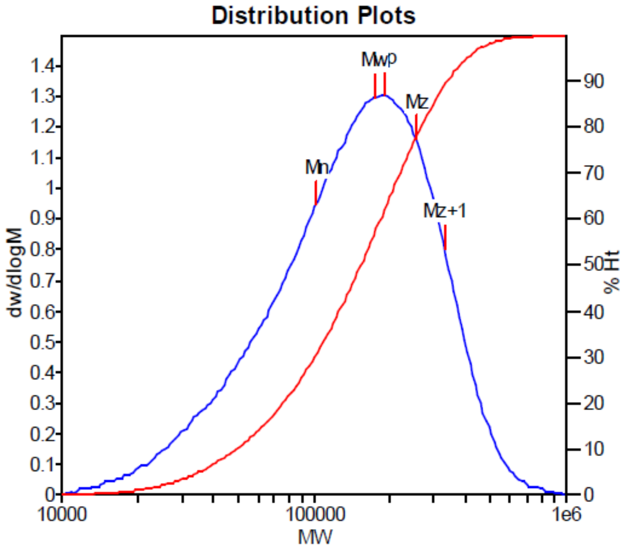

MW Averages

Peak No	Mp	Mn	Mw	Mz	Mz+1	Mv	PD
1	190789	101829	176050	254572	330807	163831	1.72888

Figure S34. Table 1, entry 2

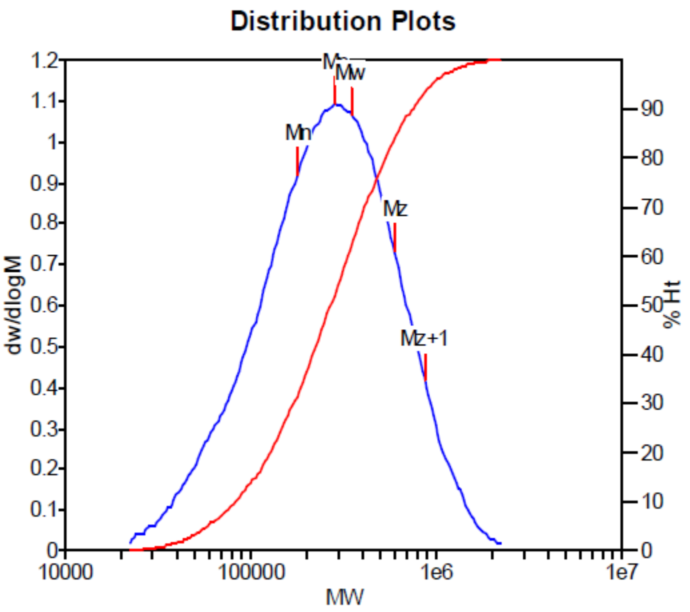

MW Averages

Peak No	Mp	Mn	Mw	Mz	Mz+1	Mv	PD
1	281952	178229	352701	599441	873226	319172	1.97892

Figure S35. Table 1, entry 3

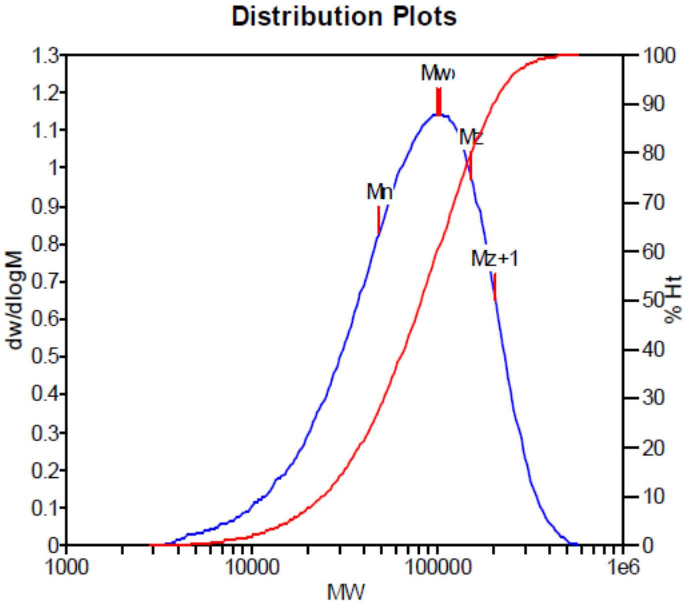

MW Averages

Peak No	Mp	Mn	Mw	Mz	Mz+1	Mv	PD
1	103575	49072	99115	153735	206112	90803	2.01979

Figure S36. Table 1, entry 4

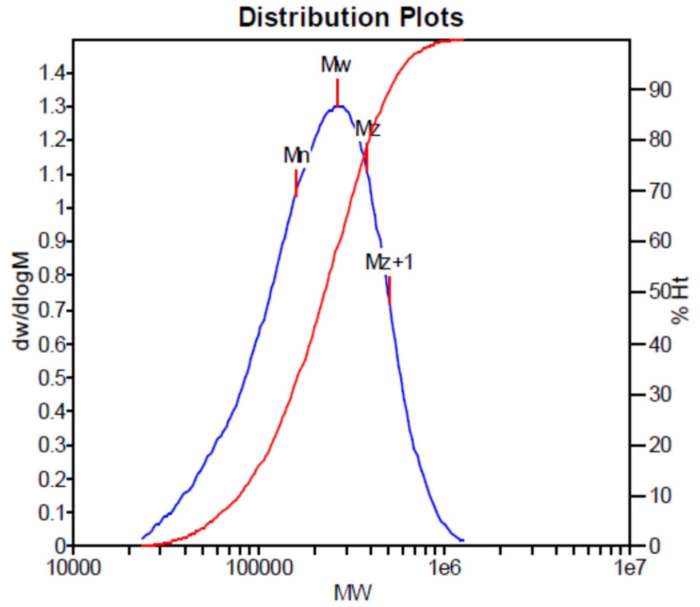

MW Averages

Peak No	Mp	Mn	Mw	Mz	Mz+1	Mv	PD
1	268497	158890	264699	384971	506416	246522	1.66593

Figure S37. Table 2, entry 1

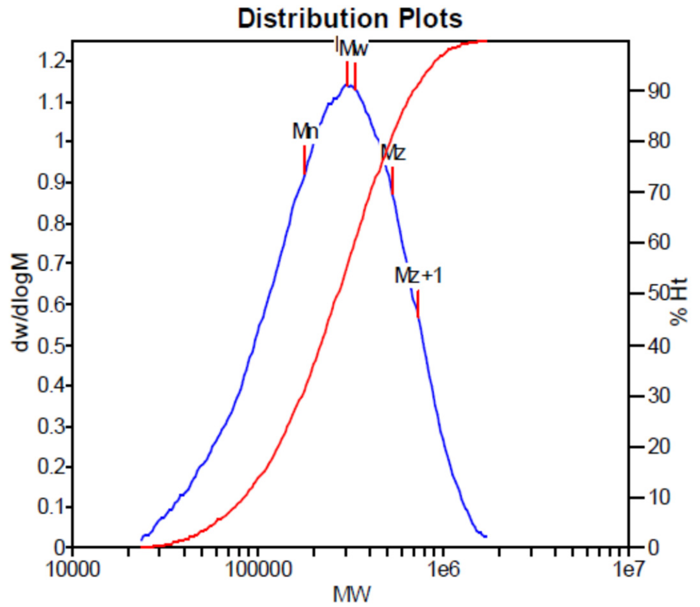

MW Averages

Peak No	Mp	Mn	Mw	Mz	Mz+1	Mv	PD
1	303411	179390	337378	534771	732226	308684	1.8807

Figure S38. Table 2, entry 2

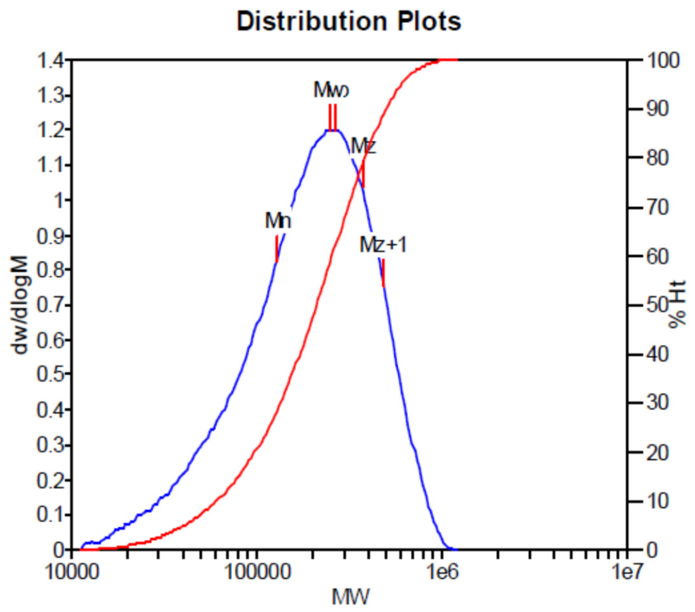

MW Averages

Peak No	Mp	Mn	Mw	Mz	Mz+1	Mv	PD
1	268608	127844	248979	373664	483550	229277	1.94752

Figure S39. Table 2, entry 3

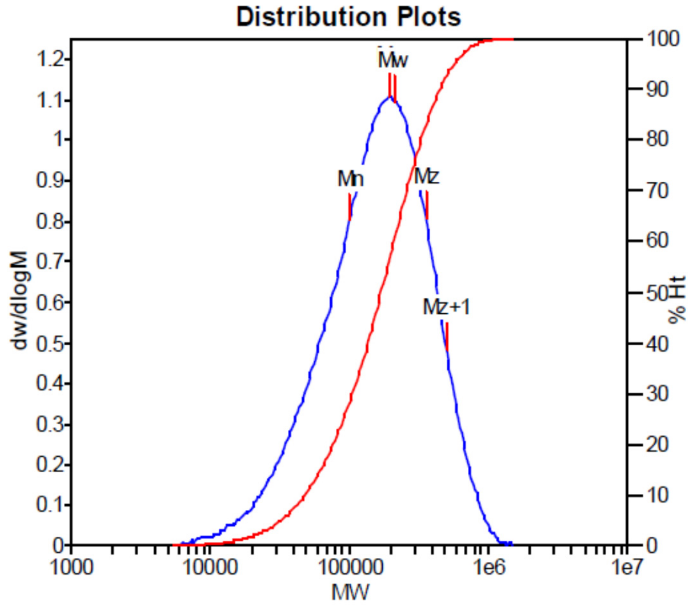

MW Averages

Peak No	Mp	Mn	Mw	Mz	Mz+1	Mv	PD
1	195508	101380	216299	357740	504813	196013	2.13355

Figure S40. Table 2, entry 4

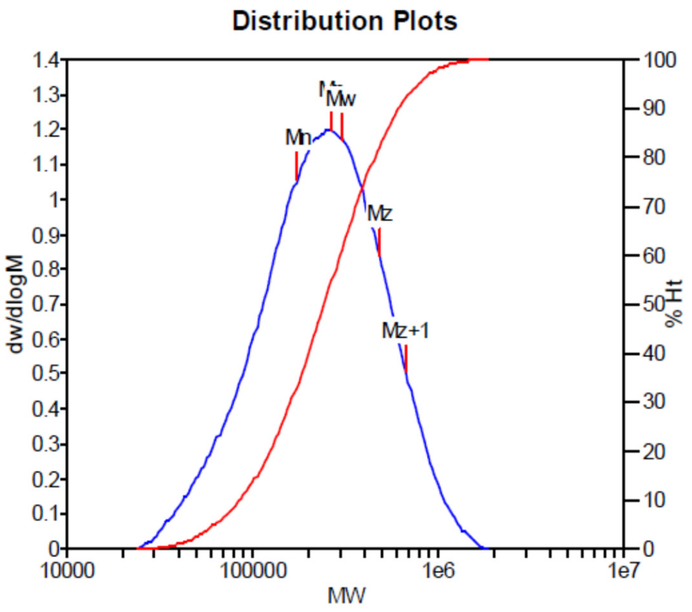

MW Averages

Peak No	Mp	Mn	Mw	Mz	Mz+1	Mv	PD
1	268497	174702	305231	480936	673967	280674	1.74715

Figure S41. Table 2, entry 5

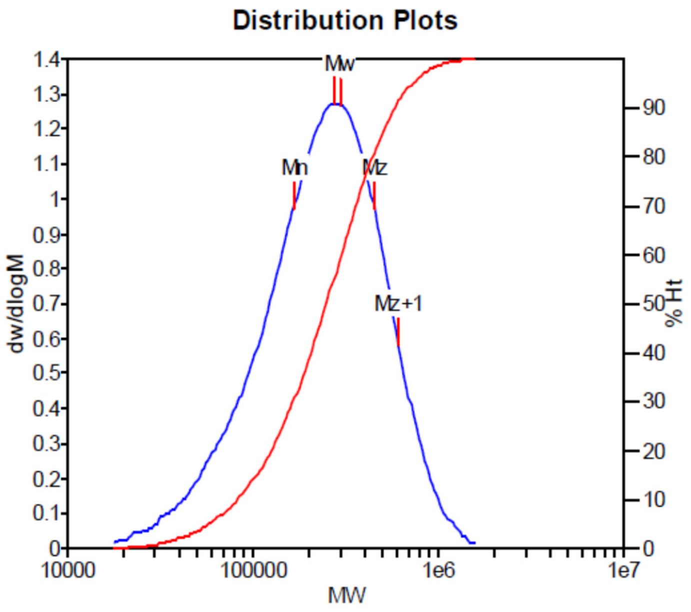

MW Averages

Peak No	Mp	Mn	Mw	Mz	Mz+1	Mv	PD
1	275142	166754	297825	451728	614476	275227	1.78601

Figure S42. Table 2, entry 6

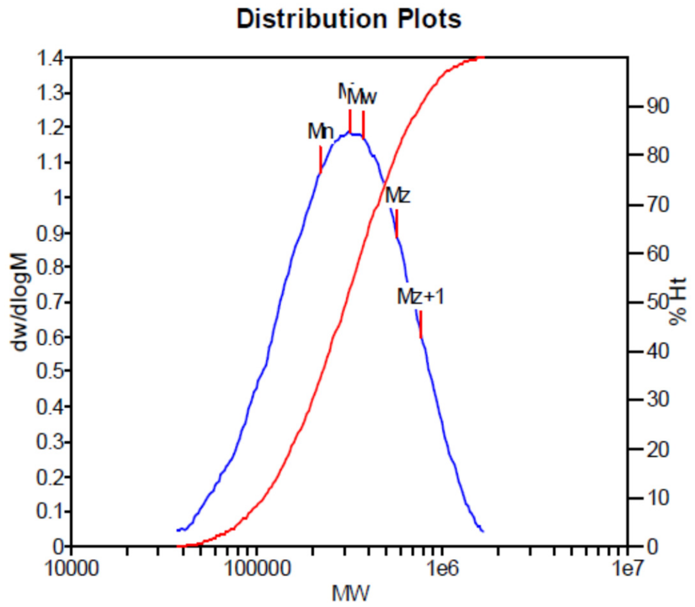

MW Averages

Peak No	Mp	Mn	Mw	Mz	Mz+1	Mv	PD
1	318617	219994	374069	571875	770984	345446	1.70036

Figure S43. Table 2, entry 7

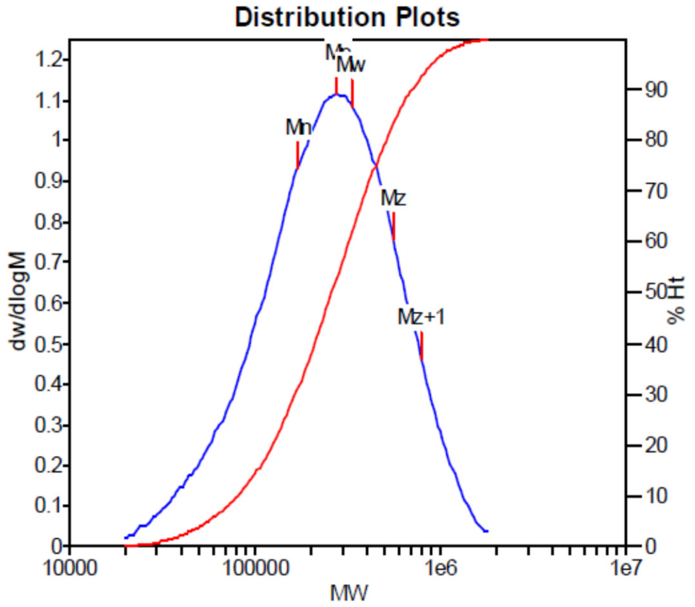

MW Averages

Peak No	Mp	Mn	Mw	Mz	Mz+1	Mv	PD
1	275142	170785	335723	557006	789748	304850	1.96576

Figure S44. Table 2, entry 8

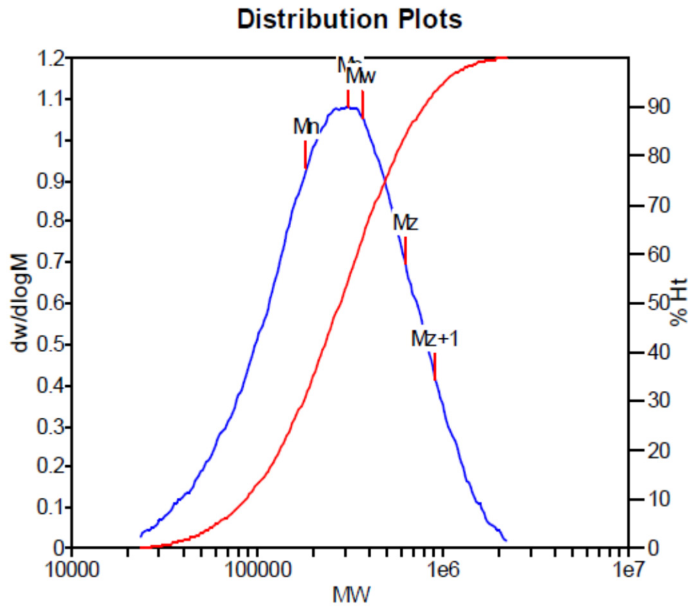

MW Averages

Peak No	Mp	Mn	Mw	Mz	Mz+1	Mv	PD
1	310921	184561	369208	633248	920706	333455	2.00047

Figure S45. Table 2, entry 9

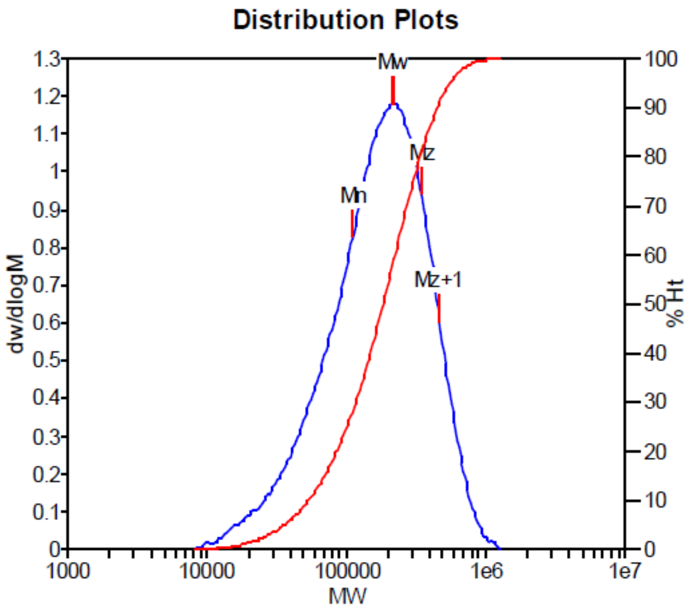

MW Averages

Peak No	Mp	Mn	Mw	Mz	Mz+1	Mv	PD
1	215582	112212	223114	347396	470074	204432	1.98833

Figure S46. Table 3, entry 1

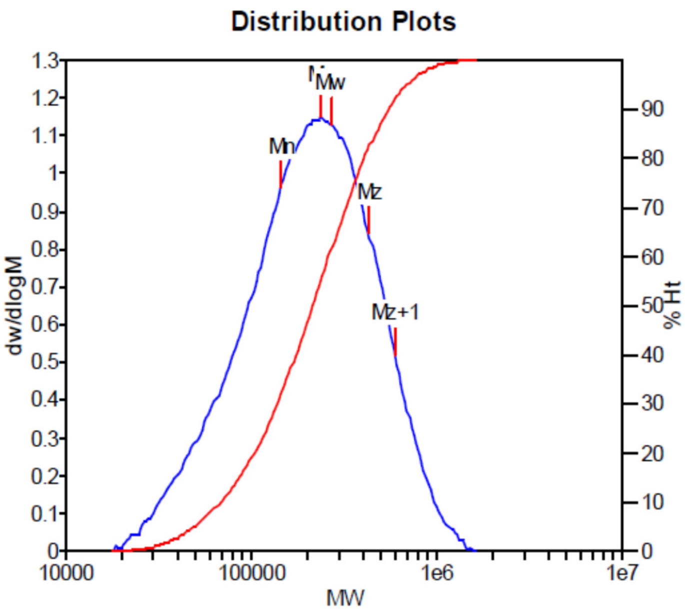

MW Averages

Peak No	Mp	Mn	Mw	Mz	Mz+1	Mv	PD
1	237717	145060	268928	429344	598441	246084	1.85391

Figure S47. Table 3, entry 2

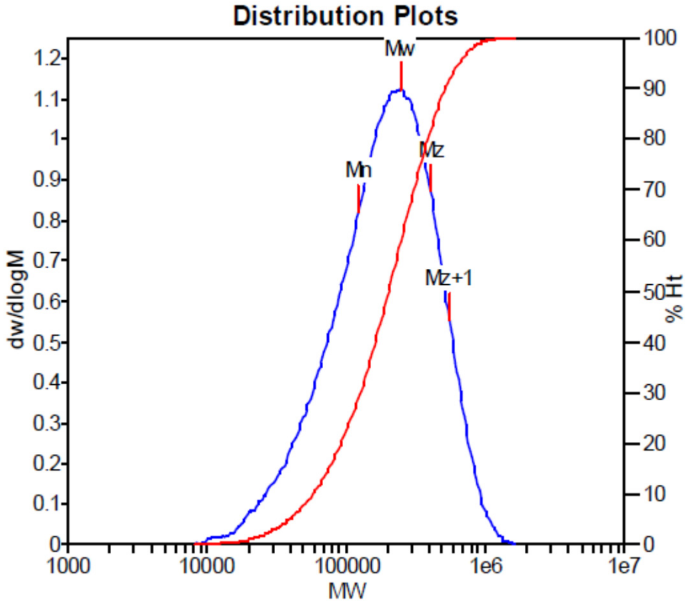

MW Averages

Peak No	Mp	Mn	Mw	Mz	Mz+1	Mv	PD
1	249622	123575	252569	405364	558367	230086	2.04385

Figure S48. Table 3, entry 3
